# Structural elucidation of a methylenation reagent of esters: synthesis and reactivity of a dinuclear titanium(iii) methylene complex†

**DOI:** 10.1039/d0sc06366e

**Published:** 2021-01-19

**Authors:** Takashi Kurogi, Kaito Kuroki, Shunsuke Moritani, Kazuhiko Takai

**Affiliations:** Division of Applied Chemistry, Graduate School of National Science and Technology, Okayama University 3-1-1 Tsushimanaka, Kita-ku Okayama 700-8530 Japan tkurogi@okayama-u.ac.jp ktakai@cc.okayama-u.ac.jp

## Abstract

Transmetallation of a zinc methylene complex [ZnI(tmeda)]_2_(μ-CH_2_) with a titanium(iii) chloride [TiCl_3_(tmeda)(thf)] produced a titanium methylene complex. The X-ray diffraction study displayed a dinuclear methylene structure [TiCl(tmeda)]_2_(μ-CH_2_)(μ-Cl)_2_. Treatment of an ester with the titanium methylene complex resulted in methylenation of the ester carbonyl to form a vinyl ether. The titanium methylene complex also reacted with a terminal olefin, resulting in olefin-metathesis and olefin-homologation. Cyclopropanation by methylene transfer from the titanium methylene proceeded by use of a 1,3-diene. The mechanistic study of the cyclopropanation reaction by the density functional theory calculations was also reported.

## Abstract 2

Transmetallation of a zinc methylene complex [ZnI(tmeda)]_2_(μ-CH_2_) with a titanium(iii) chloride [TiCl_3_(tmeda)(thf)] produced a titanium methylene complex.

## Graphical abstract

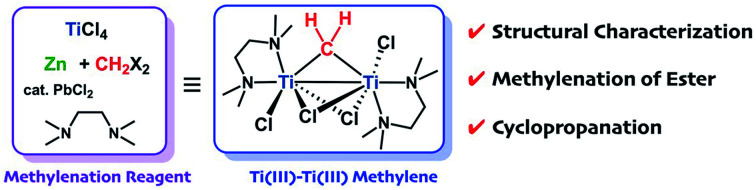

## Introduction

Since the first report of the Tebbe reagent in 1978,^1,2^ titanium methylene species^3^ have been widely investigated and utilized for Wittig-type olefination of carbonyls,^1,3*b*,3*c*,4^ C–H activation,^5^ olefin homologation,^1^ olefin metathesis^6^ and ring-opening metathesis polymerization.^7^ In the same year of Tebbe's report,^1^ Oshima and Takai also reported a methylenation reagent prepared from CH_2_Br_2_, Zn and TiCl_4_,^8^ which was modified later by Lombardo.^9^ In 1994, it turned out that the originally used zinc powder contained lead as an impurity derived from the method of metallurgy, *i.e.* pyrometallurgy,^10^ which was what catalyzed generation of the methylenation reagent (*vide infra*).^11^ It was also found that addition of *N*,*N*,*N*′,*N*′-tetramethylethylenediamine (TMEDA) to the original mixture (RCHBr_2_, Zn, cat. PbCl_2_ and TiCl_4_) dramatically changed the functional selectivity; the new reagent undergoes alkylidenation of esters,^12,13^ which cannot be achieved by the Nysted reagent [Zn_3_Br_2_(μ-CH_2_)_2_(thf)]^14^ with titanium chlorides (TiCl_4_, [TiCl_2_(O^i^Pr)_2_], [Cp_2_TiCl_2_])^15^ or the original CH_2_X_2_–Zn(Pb)–TiCl_4_ reagent without TMEDA.^8,16^

**Scheme 1:**
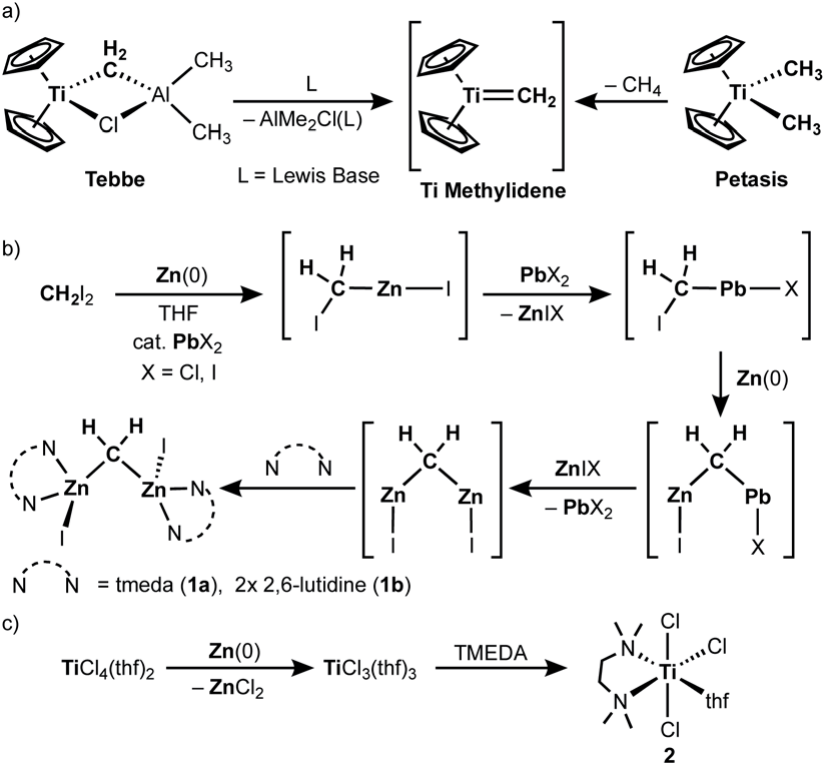
(a) Generation of a titanium methylidene from the Tebbe and Petasis reagents; (b) generation of dinuclear zinc methylene species from diiodomethane and zinc(0) catalyzed by a lead(ii) salt; (c) reduction of titanium(iv) chloride to titanium(iii) by zinc(0).

The first key step in preparing the CH_2_X_2_–Zn(Pb)–TiCl_4_ methylenation reagent involves reductive cleavage of C–X bonds by Zn(0) to form a zinc methylene species “CH_2_(ZnX)_2_”,^11,17^ which was trapped as CH_2_(SnMe_3_)_2_ upon treatment with ClSnMe_3_ (Scheme 1b).^8,16*a*^ The second reductive cleavage of a C–X bond by Zn(0) to give the zinc methylene “CH_2_(ZnI)_2_” is accelerated *via* transmetallation with a catalytic amount of a lead(ii) salt. This catalytic amount of lead crucially affects the generation of the CH_2_X_2_–Zn(Pb)–TiCl_4_ reagent.^11^ The other key step is reduction of titanium(iv) to titanium(iii) by Zn(0) (Scheme 1c),^18^ which takes place simultaneously with formation of the zinc methylene “CH_2_(ZnX)_2_”. Given the idea of a terminally bound mononuclear titanium methylene, known as “methylidene”, generated from the Tebbe and Petasis reagents (Scheme 1a),^1,19^ we believe that a transmetallation event between the zinc methylene “CH_2_(ZnI)_2_” and titanium(iii) chloride^18*b*^ should take place to generate a titanium(iii) methylidene species [(L)_*n*_Ti(=CH_2_)Cl] due to its powerful methylenation reactivity.^12,13^ To clarify the reactive species in the methylenation reagent, we have recently synthesized and isolated the zinc methylene “CH_2_(ZnX)_2_” as a dinuclear zinc μ-methylene complex, namely [ZnI(L)_*n*_]_2_(μ-CH_2_) (**1a**: L = tmeda, *n* = 1; **1b**: L = 2,6-lutidine, *n* = 2), and methylenation of ester carbonyls proceeded by mixing **1a** and [TiCl_3_(tmeda)(thf)] (**2**).^20^ Herein, we report a structural characterization of a titanium methylene complex formed by transmetallation between the zinc methylene and titanium(iii) chloride as well as methylene transfer reactions to ether carbonyls and olefins.

## Results and discussion

**Fig. 1:**
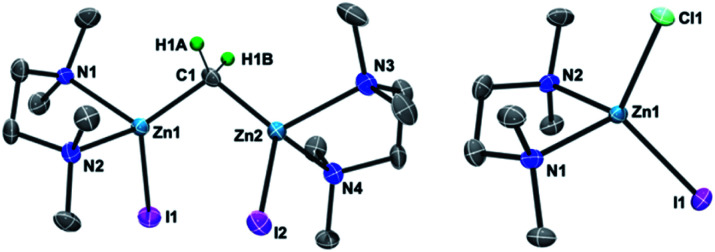
POV-ray drawing of **1a** (left) and [ZnClI(tmeda)] (right) with thermal ellipsoids at the 50% probability level. Hydrogen atoms with the exception of the methylene ligand of **1a** have been omitted for clarity.

As in our recent report,^20^ the dinuclear zinc methylene complex (**1**) was prepared by reaction of Zn(0) with CH_2_I_2_ in the presence of lead(ii) chloride^11^ and addition of TMEDA or 2,6-lutidine in THF afforded the corresponding adducts **1a** and **1b**, respectively. A solid-state structure of the TMEDA adduct **1a** (Fig. 1, left) was obtained by X-ray diffraction study of a single crystal grown in THF/hexane. Akin to the recently reported bpy^Mes^ adduct [ZnI(bpy^Mes^)]_2_(μ-CH_2_) (bpy^Mes^ = 6-Mes-2,2′-bipyridyl),^20^ the methylene ligand is bridging between two zinc iodido centers (Zn–C: 1.969(7) Å, 1.979(7) Å; Zn–C–Zn: 109.4(3)°), along with TMEDA in a bidentate coordination mode. The molecular structure of **1a** in solid-state revealed a slightly distorted *C*_2_ structure, which gave pseudo-*C*_2_ symmetric NMR spectra in solution.

**Scheme 2:**
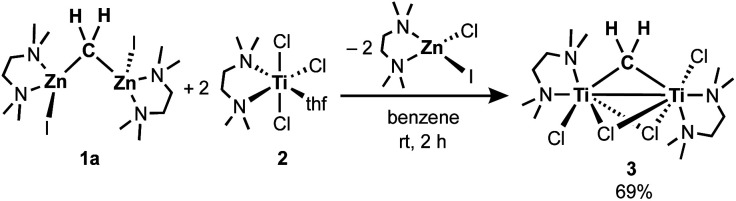
Synthesis of the titanium methylene complex **3**.

To gain insight into the transmetallation process between the zinc methylene and titanium chloride, multiple combinations of zinc methylene complexes (**1a** and **1b**) and titanium chlorides ([TiCl_4_(thf)_2_], [TiCl_4_(tmeda)], [TiCl_3_(thf)_3_], and **2**) with and without additional ligands (PR_3_, pyridine, 4-dimethylaminopyridine, and ethers) were attempted and monitored by NMR spectroscopy. In most cases, ^1^H NMR spectra revealed consumption of the zinc methylene species around −1 ppm along with formation of CH_4_ and C_2_H_4_ as well as some paramagnetic species. Interestingly, the combination of both TMEDA adducts **1a** and **2** exclusively resulted in a clean formation of a new diamagnetic titanium methylene species at 9.94 ppm,^21^ even though the originally proposed methylidene species should have a single titanium(iii) center to be paramagnetic. However, the NMR spectrum still showed a mixture with the remaining zinc methylene species in an approximately 1 : 1 ratio, which was consumed completely by addition of another equivalent of **2** (Fig. S5, ESI†).^21^ Accordingly, treatment of **1a** with two equivalents of **2** in benzene (Scheme 2) afforded complex **3** in 69% isolated yield as a reddish brown solid after removal of a zinc chlorido–iodido TMEDA complex [ZnClI(tmeda)] (Fig. 1, right), which was structurally characterized by X-ray diffraction. The NMR spectra of the isolated product **3** corroborate the methylene ligand at ^1^H: 9.45 ppm and ^13^C: 248.2 ppm (^1^*J*_CH_ = 114 Hz) along with inequivalent CH_3_ and CH_2_ resonances of TMEDA. The titanium methylene resonances of **3** are slightly down-field shifted from those of di- or trinuclear μ-methylene complexes of titanium (^1^H: 5.51–8.81 ppm, ^13^C: 188.5–253.1 ppm),^22^ but much more up-field shifted from those of mononuclear titanium methylidenes (^1^H: 11.61–12.12 ppm, ^13^C: 285.9–295.9 ppm).^23^ The methylene complex **3** is stable in solid-state at room temperature for weeks, but the ^1^H NMR resonance of the CH_2_ ligand at 9.45 ppm gradually diminished in solution at room temperature to form methane CH_4_, which was not deuterated even in THF-*d*_8_ or CD_2_Cl_2_.^21^

**Fig. 2:**
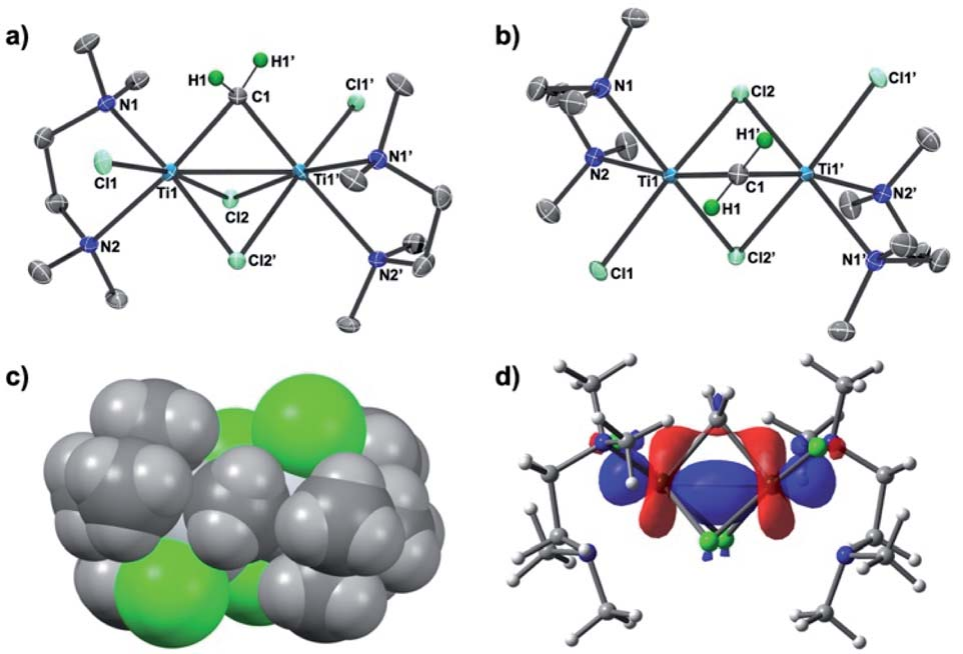
(a) Side-view of POV-ray drawing of complex **3** with thermal ellipsoids at the 50% probability level. Hydrogen atoms with the exception of the methylene ligand have been omitted for clarity; (b) top-view; (c) space-filling model of **3**; (d) molecular orbital of **3** at HOMO with the 0.04 isovalue.

To conclusively establish the connectivity in **3**, X-ray diffraction data on a single crystal grown from a concentrated THF solution were collected. As shown in Fig. 2, the solid-state structure of **3** displayed a *C*_2_ symmetric dinuclear titanium structure bridged by a methylene ligand (Ti1–C1: 2.084(6) Å; Ti1–C1–Ti1′: 78.4(3)°) and chlorides (Ti1–Cl1: 2.409(2) Å; Ti1–Cl2: 2.405(2) Å, 2.472 (2) Å). The crystal structures of the Tebbe complex [Cp_2_Ti(μ-CH_2_)(μ-Cl)AlMe_2_] have been reported by Mindiola^24*a*^ and more recently by Anwander,^24*b*^ and the Ti–CH_2_ bond length in complex **3** (2.084(6) Å) is comparable to the reported Ti–CH_2_ distances (2.095(5) Å,^24*a*^ 2.058(3) Å)^24*b*^ of the Tebbe complex. The bridging mode of the methylene ligand, where the hydrogen atoms were located from the difference map and refined isotropically, is oriented to avoid the steric repulsion with the methyl groups of TMEDA (Fig. 2c). The dinuclear methylene complex **3** is almost isostructural to the recently reported dinuclear chromium(iii) alkylidene complexes with TMEDA ligands, [CrCl(tmeda)]_2_(μ-CHR)(μ-Cl)_2_ (R = SiMe_3_, GeMe_3_, SnMeCl_2_), but those dinuclear chromium(iii) alkylidene complexes could be enforced to have a *C*_s_ symmetry due to the bulky substituents on the bridging alkylidenes.^26^ In contrast to the reported dinuclear Ti(iv)–Ti(iv) methylene complexes,^22^ the molecular structure of the Ti(iii)–Ti(iii) methylene complex **3** showed a short Ti–Ti distance of 2.634(2) Å, which is much shorter than the sum of van der Waals radii.^25^ The density functional theory (DFT) calculations of **3** in singlet (**13**) revealed a Ti–Ti bonding interaction (2.637 Å) at the HOMO (Fig. 2d) along with the Wiberg bond order index 0.96, while the optimized structure in triplet (**33**) has a much longer Ti–Ti distance (3.224 Å).

**Scheme 3:**
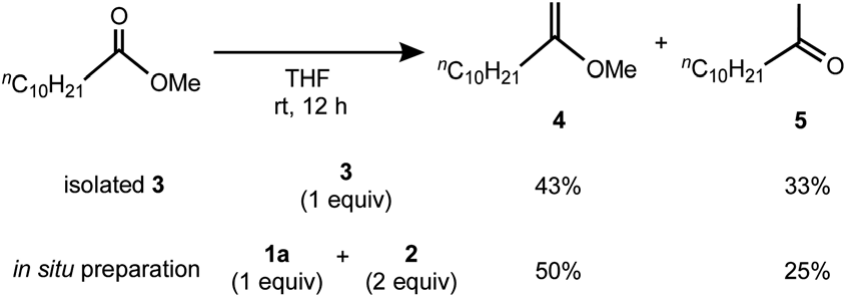
Methylenation of methyl undecanoate by isolated **3** and *in situ* preparation of **3**.

Having the methylene complex **3** in hand, we resorted to demonstrating methylenation of esters,^12^ which can be achieved by titanium methylidene species such as the Tebbe reagent.^4*a*^ As shown in Scheme 3, methylenation of methyl undecanoate with one equivalent of **3** in THF smoothly proceeded in 76% yield to give a mixture of 2-methoxy-1-decene (**4**) and its hydrolysis product **5**,^27^ comparable to that with the reagent prepared *in situ* from **1a** and **2** in a 1 : 2 ratio (**4**: 50%; **5**: 25%). In contrast, no methylenation products **4** or **5** were observed without TMEDA, *e.g.* a mixture of the 2,6-lutidine adduct **1b** and [TiCl_3_(thf)_3_],^20^ implying the necessity of TMEDA to generate the reactive species for methylenation of esters. In addition, methylenation of cyclic esters with **3** could proceed to afford cyclic vinyl ethers, but some ring-opening and oligomerization products were also formed.^21^

**Scheme 4:**
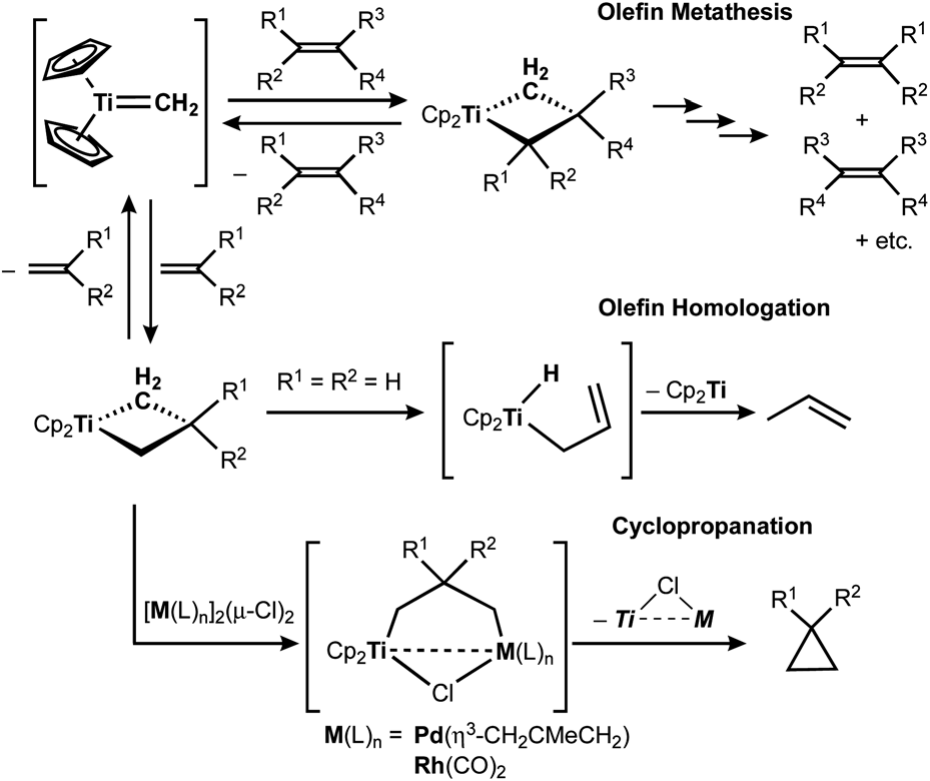
Reversible [2+2]-cycloaddition of olefin to titanium methylidene^1,28^ and olefin-metathesis,^6,7^ olefin-homologation,^1^ and formation of cyclopropane from titanacyclobutanes.^32,33^

**Scheme 5:**
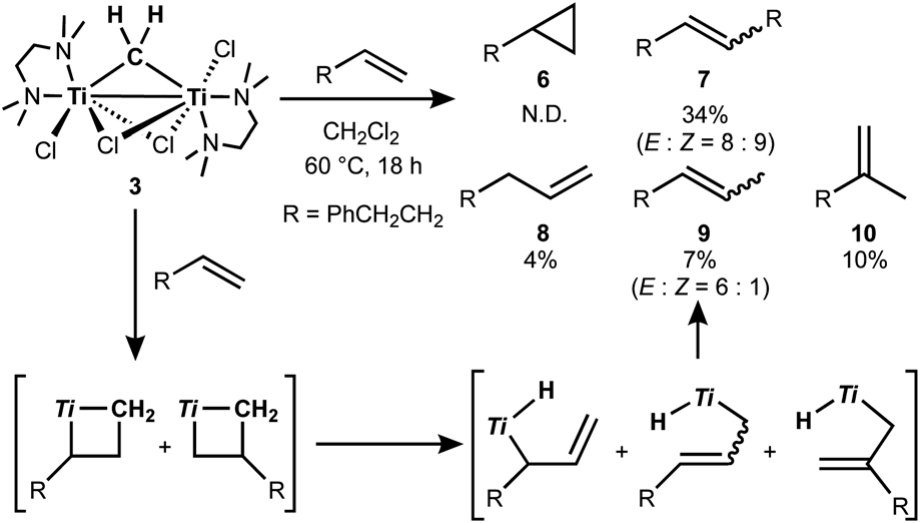
Olefin-metathesis and homologation of terminal olefin by **3** and the proposed mechanism (Ti = TiCl(tmeda)).

The titanocene methylidene generated from the Tebbe or Petasis reagents reacts with olefins to form titanacyclobutanes reversibly (Scheme 4),^1,28^ resulting in olefin-metathesis.^6,7^ Tebbe and Parshall also found that titanacyclobutanes can undergo β-H elimination and formation of olefin-homologation products.^1^ In contrast to other metallacyclobutanes,^29^ Grubbs and co-workers pointed out the difficulty in promoting reductive elimination of cyclopropane from the mononuclear titanium(iv) metallacyclobutane due to formation of a thermodynamically unfavored titanium(ii) product,^30,31^ unless assisted by oxidation with I_2_ ^32^ or formation of metal–metal interacting heterobimetallic species.^33^ In fact, formation of the cyclopropanation product (**6**) by treatment of a terminal olefin, 4-phenyl-1-butene, with our dinuclear titanium methylene **3** was not observed. Instead, NMR and GC analyses revealed formation of the corresponding olefin-metathesis (**7**: 34%)^34^ and olefin-homologation products (**8**: 4%; **9**: 7%; **10**: 10%).^21^ As illustrated in Scheme 5, each olefin-homologation product should be formed *via* the corresponding titanacyclobutane and allyl-hydrido intermediates.

**Scheme 6:**
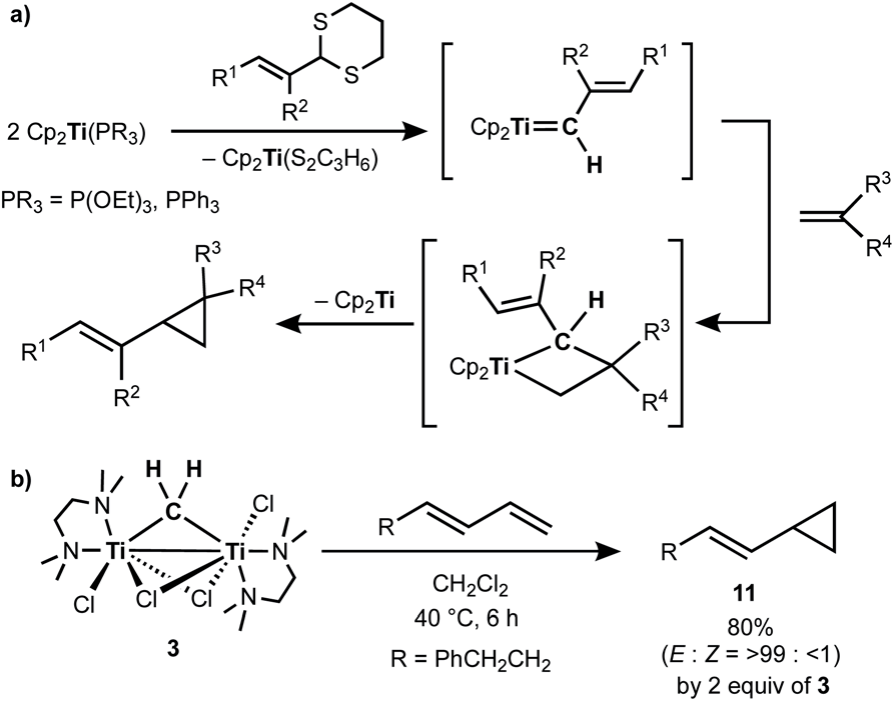
(a) Vinylcyclopropanation of terminal olefins by titanocene alkylidenes;^35^ (b) cyclopropanation of a 1,3-diene by complex **3**.

Takeda and co-workers reported a successful example of cyclopropanation by a combination of titanocene allylidenes and terminal olefins (Scheme 6a),^35^ while olefin-homologation of terminal olefins was observed by use of titanocene alkylidenes without α-vinyl groups.^36^ Inspired by Takeda's work on the vinylcyclopropanation system, we employed a 1,3-diene for the cyclopropanation reaction by methylene transfer from complex **3**. Treatment of a 1,3-diene, (*E*)-6-phenyl-1,3-hexadiene, with **3** at room temperature in CH_2_Cl_2_ resulted in selective formation of an (*E*)-vinyl cyclopropane (**11**) in 49% yield (Scheme 6b). More conversion of the 1,3-diene to cyclopropane **11** (80% yield) was achieved by further addition of **3** (2 equiv.) and gentle heating at 40 °C. However, various olefin-metathesis products from the 1,3-diene as well as a small amount of the *Z*-isomer of **11** were formed by performing the reaction at 80 °C (Fig. S12, ESI†).

**Fig. 3:**
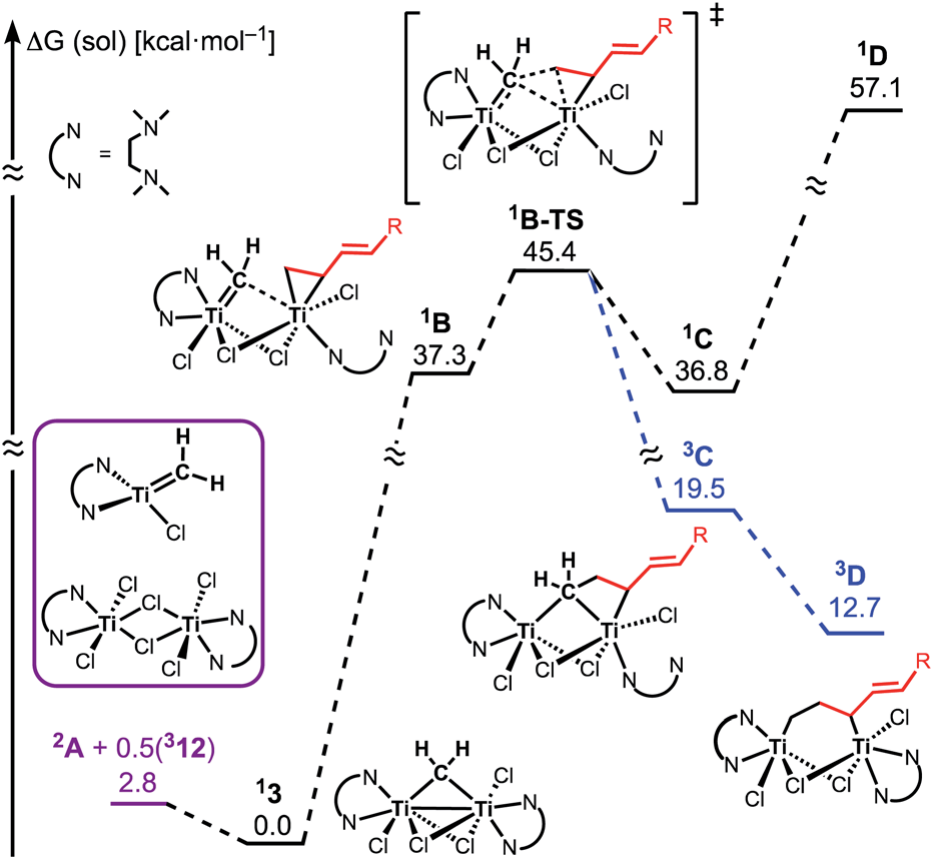
Free energy profiles for insertion of 1,3-diene to Ti–CH_2_. The superscripted number on the left top represents the spin-multiplicity of each titanium species.

**Fig. 4:**
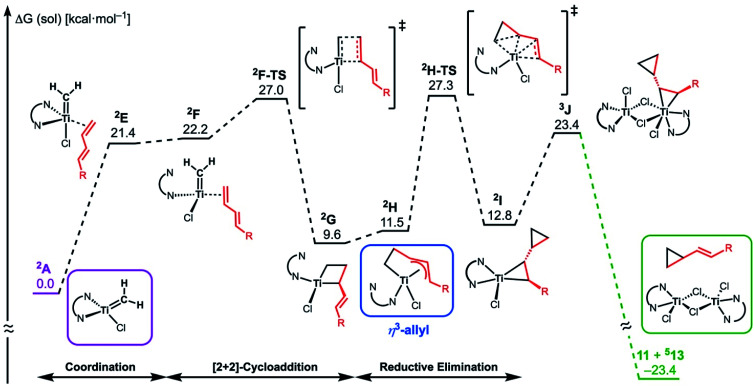
Free energy profile for the most plausible pathway of cyclopropanation of 1,3-diene *via* a mononuclear titanium(iii) methylidene (**A**). The free energy values are presented based on the methylidene species **A**. The superscripted number on the left top represents the spin-multiplicity of each titanium species.

**Fig. 5:**
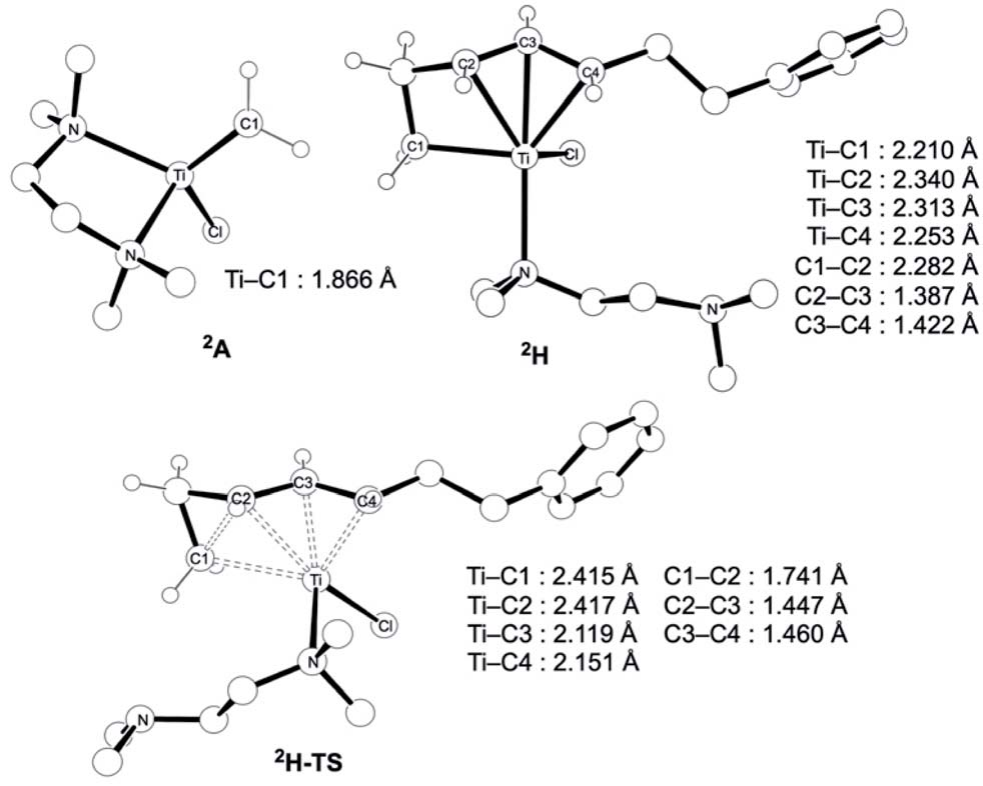
Optimized structures of a titanium(iii) methylidene species **2A** (left top), η^3^-allyl intermediate **2H**, and a transition state of cyclopropanation **H-TS** (bottom).

The similar reactivity of our titanium methylene **3** with titanocene alkylidenes raised the question of whether our dinuclear Ti(iii)–Ti(iii) system remains in the dinuclear structure during the reaction^37^ or generates a mononuclear titanium methylidene. Unfortunately, experimental observation of the dinuclear or mononuclear titanium methylidene species in the reaction system has not been achieved and we decided to carry out the mechanistic study by quantum chemical calculations based on DFT. The vinylcyclopropanation reaction system by complex **3** and a 1,3-diene was employed to gain insight into the reaction selectivity of cyclopropanation. We first examined whether the 1,3-diene forms a new C–C bond *via* insertion to the Ti–C bond in the dinuclear complex **3** (Fig. 3) or *via* [2+2]-cycloaddition with a mononuclear titanium methylidene species [Ti=CH_2_] (**A**) (Fig. 4). Despite the dinuclear structure of **3** being 2.8 kcal mol^−1^ lower in relative free energy than generation of **A** and [TiCl_2_(tmeda)](μ-Cl)_2_ (**12**) (Fig. S14, ESI†), the dinuclear 1,3-diene adduct **B**, in which the [TiCH_2_Ti] unit is better described as a semi-bridging titanium methylidene (Ti–CH_2_: 1.886 Å, 2.546 Å), could be located at 37.3 kcal mol^−1^ (Fig. 3).^38^ The transition state of the insertion step (**B-TS**) was also found with a very high reaction barrier of 45.4 kcal mol^−1^ leading to dimetallacycle species **C** and **D**. In contrast, the mononuclear 1,3-diene adduct **E** was located at 21.4 kcal mol^−1^ from **A**. Upon [2+2]-cycloaddition as illustrated in Fig. 4, the TMEDA ligand changes its coordination mode into a κ^1^-fashion (**F**) to traverse the transition state **F-TS**. The resulted titanacyclobutane intermediate **G** undergoes hapticity change of a vinyl group on the α-position to transform into an η^3^-allyl species **H**. To afford the corresponding cyclopropane **11**, reductive elimination from the mononuclear titanium(iii) metallacyclobutane should take place, requiring formation of a thermodynamically unfavored titanium(i) species. The π-allylic interaction in **H** allows reductive ring-closing elimination in **H-TS** to maintain the titanium(iii) character (Fig. 5). As a result, this back-bonding interaction lowers the reaction barrier of the reductive elimination process to give the cyclopropanation product selectively rather than undergoing metathesis or β-H elimination process as in the reaction of terminal olefins.^39^ Note that a positional isomer of **G**, β-vinyl titanacyclobutane (**G′**) given by [2+2]-cycloaddition in the other fashion, is also a conceivable intermediate but cannot form a similar π-allyl configuration due to its strained structure for coordination to the titanium center.^40^ The mononuclear complex **I** may bind [TiCl_3_(tmeda)] to form a dinuclear chloride (**J**) and then eliminate cyclopropane **11** and a titanium(ii) chloride dimer (**13**) rather than formation of unlikely “TiCl(tmeda)”. Although a mononuclear titanium(ii) chloride [TiCl_2_(tmeda)_2_] is kinetically stable,^30^ the titanium(ii) chloride dimer [TiCl(tmeda)]_2_(μ-Cl)_2_ (**13**) can readily undergo disproportionation to titanium(iii) chloride and some low-valent byproducts.^30*a*,31^ In fact, a dinuclear mixed-valent Ti(ii)–Ti(iii) chloride [TiCl(tmeda)]_2_(μ-Cl)_3_ (Fig. S15, ESI†), which has been reported by Gambarotta from thermal decomposition of [TiCl_2_(tmeda)_2_],^30*a*^ was reproducibly observed in our cyclopropanation reaction system.

## Conclusions

We have shown a transmetallation pathway between a zinc methylene complex [ZnI(tmeda)]_2_(μ-CH_2_) and titanium(iii) chloride, resulting in formation of a dinuclear titanium methylene [TiCl(tmeda)]_2_(μ-CH_2_)(μ-Cl)_2_. The solid-state structure showed the first example of a dinuclear Ti(iii)–Ti(iii) methylene complex. Methylene transfer reactions to ester, terminal olefin and 1,3-diene have been demonstrated. The powerful methylenation reactivity and mechanistic study both propose generation of a titanium(iii) methylidene species.

## Conflicts of interest

There are no conflicts to declare.

## Supplementary Material

SC-012-D0SC06366E-s001

SC-012-D0SC06366E-s002
