# Supplementary material for: Structural elucidation of a methylenation reagent of esters: synthesis and reactivity of a dinuclear titanium(iii) methylene complex
Source: Chem Sci. 2021 Jan 19;12(10):3509–15. doi: 10.1039/d0sc06366e (PMC8179466; doi:10.1039/d0sc06366e)
Supplement: SC-012-D0SC06366E-s001 [file SC-012-D0SC06366E-s001.pdf]

Electronic Supplementary Material (ESI) for Chemical Science.
This journal is © The Royal Society of Chemistry 2021

Structural Elucidation of a Methylenation Reagent of Esters: Synthesis and Reactivity of a Dinuclear Titanium(III) Methylene Complex

Takashi Kurogi,[†,*] Kaito Kuroki,[†] Shunsuke Moritani[†] and Kazuhiko Takai[†,*]

[†]*Division of Applied Chemistry, Graduate School of Natural Science and Technology, Okayama University, 3-1-1 Tsushimanaka, Kita-ku, Okayama 700-8530, Japan.*

Table of Contents

**General Procedure**	S3
**Improved Synthesis of [ZnI(tmeda)]$_2$(μ-CH$_2$) (1a)**	S4
**Improved Synthesis of TiCl$_3$(tmeda)(thf) (2)**	S4-S5
**Synthesis of [TiCl(tmeda)]$_2$(μ-CH$_2$)(μ-Cl)$_2$ (3)**	S5-S8
**NMR Reaction of 1a and 2**	S9-S11
**Stability Testing of 3**	S12-S13
**Methylenation of Methyl Undecanoate by 3**	S14-S15
**Methylenation of γ-Undecanolactone by 3**	S16-S17
**Reaction of 4-Phenyl-1-butene with 3**	S18-S19
**Cyclopropanation of 6-Phenylhexa-1,3-diene by 3**	S20-S21
**FID-GC and GC-MS Analyses**	S22-S23
**X-ray Crystallography**	S24-S26
Crystallographic data of **1a**	S27
Crystallographic data of ZnClI(tmeda)	S28
Crystallographic data of **2**	S29
Crystallographic data of [TiCl$_2$(tmeda)]$_2$(μ-Cl)$_2$	S30
Crystallographic data of **3**	S31

Crystallographic data of $[TiCl(tmeda)]_2(\mu\text{-}Cl)_3$	S32
**Computational Details**	S33-S34
**Cartesian Coordinates of the Optimized Geometries**	S35-S50
**References**	S51-S52

General Procedure

All operations were performed in a Vac. glove box under a dinitrogen atmosphere or using standard Schlenk techniques under an argon atmosphere unless otherwise stated. Anhydrous hydrocarbon solvents (hexane, benzene, toluene), stabilizer-free ethereal solvents (Et_2O, THF) and dichloromethane (stabilized with amylene) were purchased from FUJIFILM Wako Pure Chemical Corporation. All anhydrous solvents were stored over activated 4 Å molecular sieves and degassed by bubbling argon. Benzene-d_6 (Cambridge Isotope Laboratories) and THF-d_8 (Cambridge Isotope Laboratories) were dried and degassed over a potassium mirror prior to use. Dichloromethane-d_2 (Cambridge Isotope Laboratories) was dried over activated 4Å molecular sieves overnight and degassed by freeze-pump-thaw cycles. Molecular sieves were activated under vacuum at 200 °C. *N*,*N*,*N*',*N*'-Tetramethyethylenediamine (TMEDA) was purchased from TCI Chemicals and distilled over calcium hydride. $TiCl_3(thf)_3$,[1] $[ZnI(2,6\text{-lutidine})_2]_2(\mu\text{-}CH_2)$ (**1b**),[2] 6-phenylhexa-1,3-diene,[3] and authentic samples of **7**,[4] **8**,[5] **9**,[6] and **10**[7] were prepared according to the reported procedures. All other chemicals were purchased from commercial sources and degassed before being used. ^{1}H, ^{13}C and HMQC NMR spectra were recorded on a JEOL ECS 400 MHz spectrometer. ^{1}H and ^{13}C NMR chemical shifts are reported referenced to the internal residual proton or carbon resonances of C_6D_6 (^{1}H: 7.16 ppm, ^{13}C: 128.06 ppm), C_4D_8O (^{1}H: 3.58 or 1.73 ppm, ^{13}C: 67.21 or 25.31 ppm), $CDCl_3$ (^{1}H: 7.26 ppm, ^{13}C: 77.16 ppm), and CD_2Cl_2 (^{1}H: 5.32 ppm, ^{13}C: 53.84 ppm). Elemental analyses were performed on a Perkin Elmer 2400II and an Elementar VarioMicroCube. High-resolution mass spectra (HRMS) were measured by fast atom bombardment (FAB) using a double focusing magnetic sector mass spectrometer (JEOL JMS-700 MStation FAB-MS). Gel permeation chromatography (GPC) was performed by a LC-9210NEXT (Japan Analytical Industry Co., Ltd.) equipped with two JAIGEL-2HR columns (Japan Analytical Industry Co., Ltd.).

Improved Synthesis of $[ZnI(tmeda)]_2(\mu\text{-}CH_2)$ (1a)

Synthesis of $[ZnI(tmeda)]_2(\mu\text{-}CH_2)$ (**1a**) has been previously reported by reaction of CH_2I_2 and zinc powder in the presence of lead(II) chloride in THF and addition of TMEDA.[2] However, an off-white viscous solid is often obtained by this method after work-up probably due to an excessive amount of TMEDA. Addition of a stoichiometric amount of TMEDA to the isolated 2,6-lutidine adduct **1b** is more suitable to obtain a pure white solid of **1a** as described below.

To a colorless solution of **1b** (5.28 g, 6.38 mmol) in THF (15 mL) was added TMEDA (1.46 g, 12.6 mmol) at room temperature, resulting in a slight color change to pale yellow. The reaction mixture was stirred at room temperature for 30 minutes. All volatile materials were removed under vacuum. The white residue was washed with hexane (2 x 10 mL) and dried under vacuum to yield $[ZnI(tmeda)]_2(\mu\text{-}CH_2)$ (**1a**) as a white solid (3.62 g, 5.74 mmol, 90% yield). The ^1H NMR spectrum of the obtained white solid in dichloromethane-d_2 showed the same spectrum with **1a** reported in our previous paper.[2] Colorless crystals suitable of **1a** for X-ray analysis were grown from a concentrated THF solution layered with hexane at −20 °C.

Improved Synthesis of $[TiCl_3(tmeda)(thf)]$ (2)

Synthesis of $TiCl_3$(tmeda)(thf) (**2**) has been previously reported by reduction of $TiCl_4$ with zinc powder in the presence of TMEDA in THF.[8] However, isolation of **2** is often hampered by a similar solubility of the byproduct $ZnCl_2$(tmeda). Ligand exchange of isolated $TiCl_3(thf)_3$, prepared from $(TiCl_3)_3\cdot AlCl_3$ (or prepared *in situ* by reduction of $TiCl_4(thf)_2$ with aluminum powder in THF), is more suitable to obtain pure $TiCl_3$(tmeda)(thf) (**2**) as described below.

To a light blue suspension of $TiCl_3(thf)_3$ (7.43 g, 20.1 mmol) in THF (30 mL) was added TMEDA (3.30 mL, 22.2 mmol) at room temperature. The reaction mixture was stirred at 50 °C for 3 hours and turned into a deep blue solution. The reaction mixture

was cooled down to room temperature and added hexane (20 mL), resulting in a blue suspension. The supernatant was removed by cannula-transfer and the blue residue was dried under vacuum to yield **2** as a blue crystalline solid (6.50 g, 19.0 mmol, 95% yield). Blue crystals of **2** suitable for X-ray analysis were grown from a concentrated THF solution layered with hexane at room temperature. An X-ray analysis of the obtained blue crystals showed the same unit cell with $TiCl_3$(tmeda)(thf) reported in the previous paper.[8] Pale brown crystals of $[TiCl_2(tmeda)]_2(\mu\text{-}Cl)_2$ were also grown from a concentrated CH_2Cl_2 solution layered with hexane at room temperature. Anal. Calcd. for $C_{12}H_{32}N_4Cl_6Ti_2$: C, 26.65; H, 5.96; N, 10.36. Found: C, 26.51; H, 6.01; N, 10.23.

Synthesis of $[TiCl(tmeda)]_2(\mu\text{-}CH_2)(\mu\text{-}Cl)_2$ (3)

To a suspension of **2** (4.39 g, 12.9 mmol) in benzene (20 mL), changing in color by dissociation of THF in benzene, was added **1a** (3.79 g, 6.01 mmol) as solid at room temperature. The reaction mixture was stirred for 2 hours, resulting in a reddish brown suspension. The reddish brown solid was collected by filtration on a glass frit and washed with benzene (2 x 10 mL), THF (2 x 15 mL) and hexane (20 mL). The brown residue was dried under vacuum to yield **3** as a reddish brown solid (2.01 g, 4.15 mmol, 69% yield). Brown crystals of **3** suitable for X-ray analysis were grown from a concentrated THF solution layered with hexane at −20 °C. Colorless crystals of ZnClI(tmeda) were also grown from the filtrate (washing with benzene) along with black insoluble materials from decomposition of titanium species.

^{1}H NMR (400 MHz, THF-d_8, 25 °C): δ 9.45 (s, 2H, TiCH_2Ti), 4.29 (s, 6H, TMEDA-CH_3), 3.02 (br t, J_{HH} = 11 Hz, 2H, TMEDA-CH_2), 2.39 (s, 6H, TMEDA-CH_3), 1.99 (br dd, J_{HH} = 4 Hz, 10 Hz, 2H, TMEDA-CH_2), 1.84 (s, 6H, TMEDA-CH_3), 1.66 (s, 6H, TMEDA-CH_3), 1.56 (br m, 2H, TMEDA-CH_2). ^{13}C{^{1}H} NMR (101 MHz, dichloromethane-d_2, 25 °C) δ 248.2 (TiCH_2Ti), 58.3 (TMEDA), 55.5 (TMEDA), 51.4 (TMEDA), 50.7 (TMEDA), 49.2 (TMEDA). Anal. Calcd. for $C_{13}H_{34}N_4Cl_4Ti_2$: C, 32.26;

H, 7.08; N, 11.58. Found: C, 32.41; H, 7.32; N, 11.49.

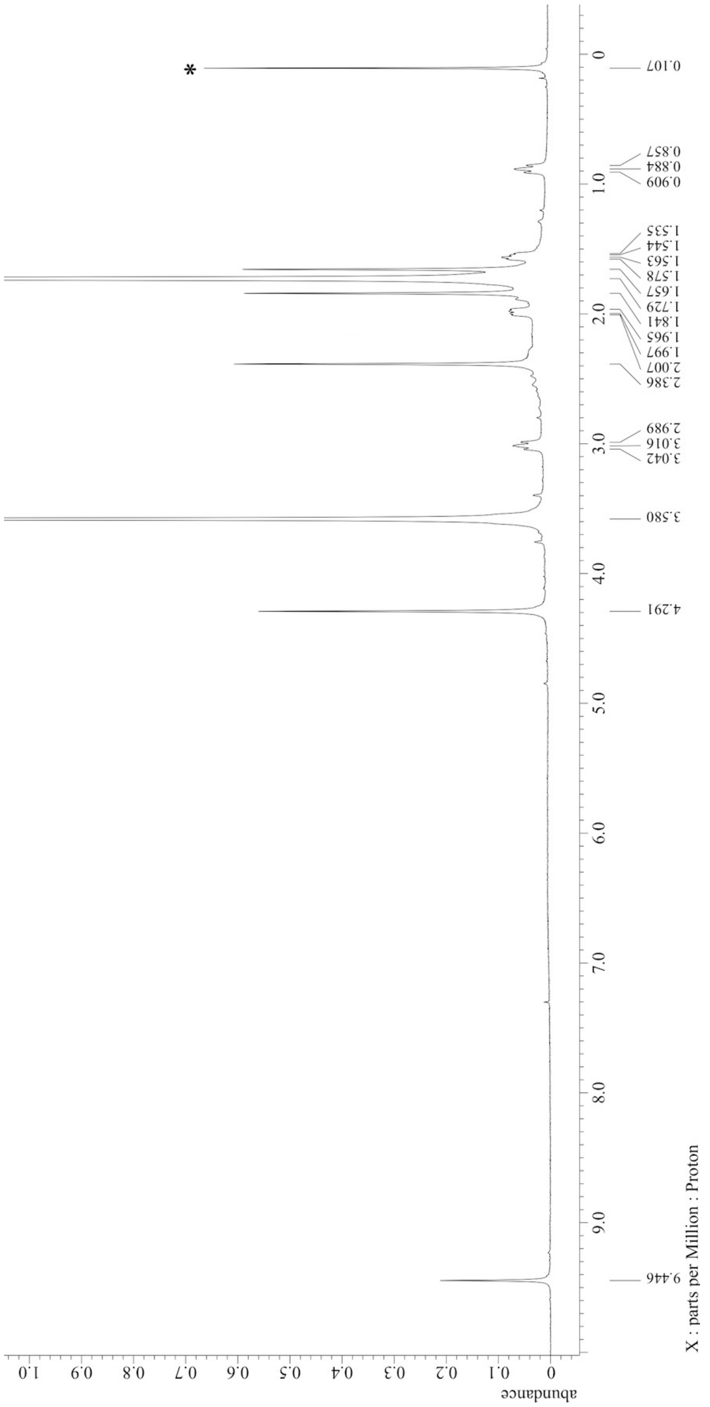

Fig. S1 ^{1}H NMR spectrum of **3** (400 MHz, in THF-d_8, at 25 °C). *: silicone grease.

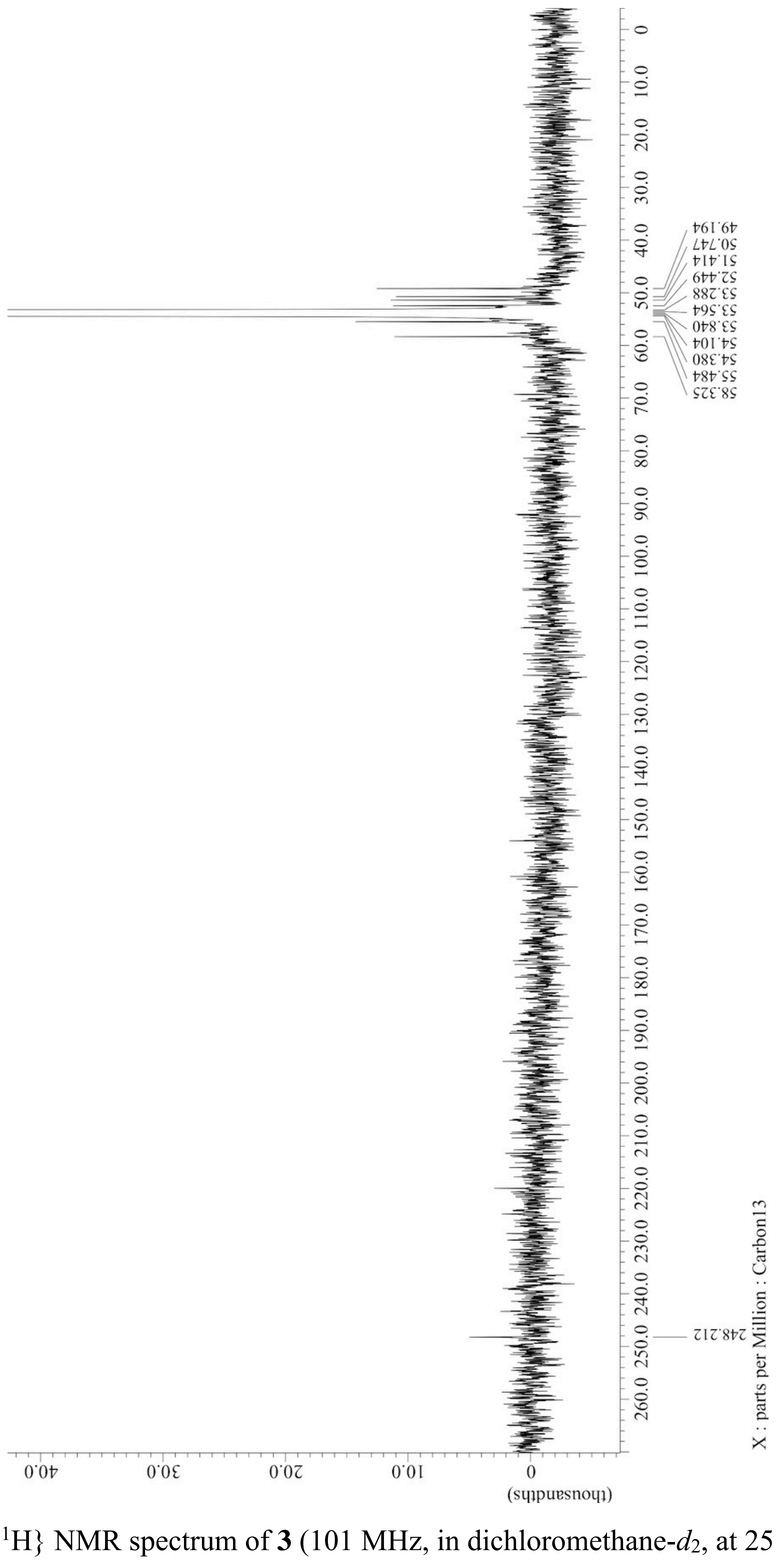

Fig. S2 $^{13}C\{^{1}H\}$ NMR spectrum of **3** (101 MHz, in dichloromethane-d_2, at 25 °C).

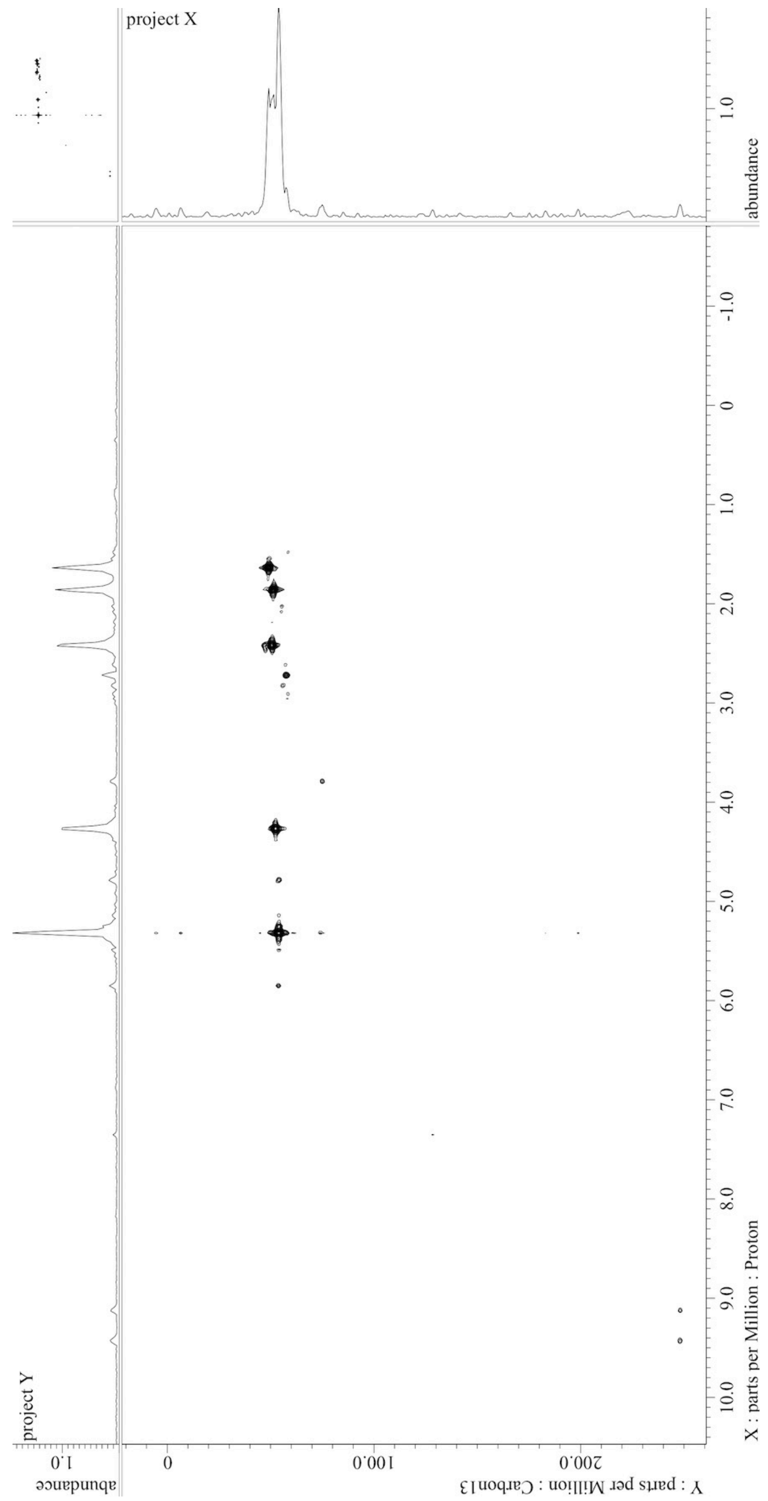

Fig. S3 ^{1}H-^{13}C HMQC NMR spectrum of **3** (400 MHz, in dichloromethane-d_2, at 25 °C).

NMR Reaction of 1a and 2

In a J. Young valve NMR tube, complex **1a** (10 mg, 15.8 μmol) and **2** (5.5 mg, 16.1 mmol) were suspended in benzene-d_6 (ca. 0.6 mL). The reaction mixture readily changed to a brown suspension. The ^{1}H NMR spectrum (Fig. S4) after 10 min at room temperature showed formation of complex **3** at 9.96 ppm for the Ti-CH_2-Ti moiety. However, some zinc methylene species around −1 ppm still remained. Addition of another equivalent of **2** (6.1 mg, 17.8 μmol) resulted in consumption of the remaining zinc methylene species (Fig. S5). As a result, we concluded that formation of complex **3** requires two equivalents of **2** to **1a**.

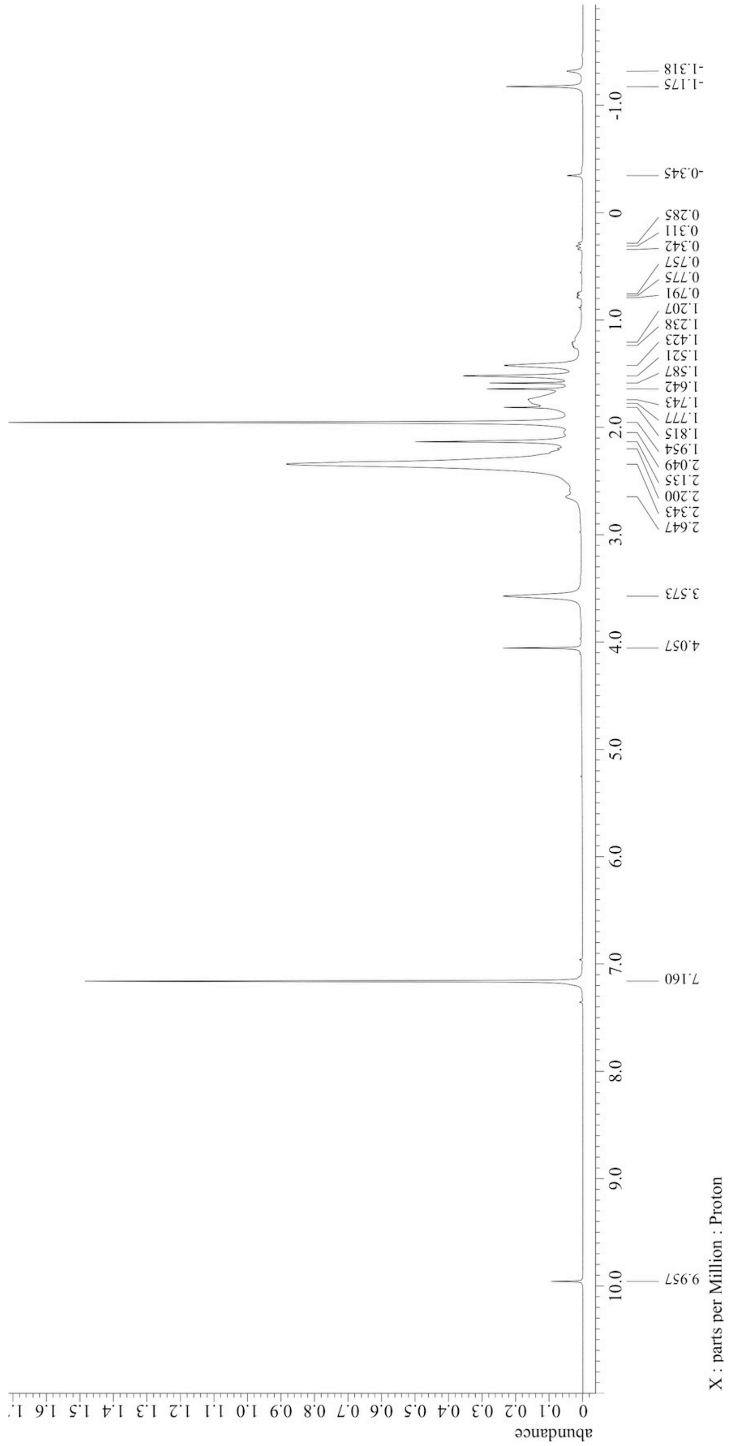

Fig. S4 ^{1}H NMR spectrum of a mixture of **1a** and **2** in a 1:1 ratio (400 MHz, in benzene-d_6, at 25 °C).

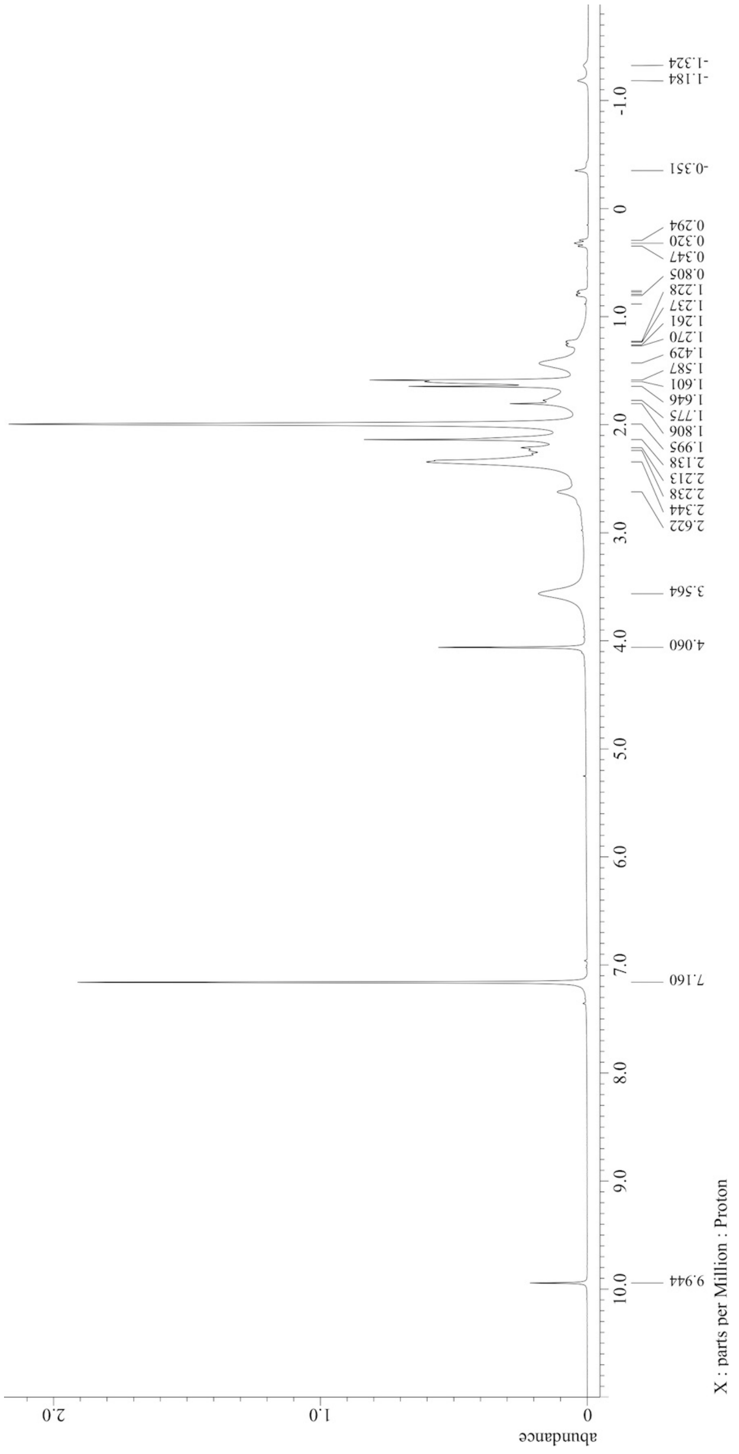

Fig. S5 ^{1}H NMR spectrum of a mixture of **1a** and **2** in a 1:2 ratio (400 MHz, in benzene-d_6, at 25 °C).

Stability Testing of 3

Complex **3** is stable in solid-state as a reddish brown solid at room temperature under an inert atmosphere. Complex **3** is poorly soluble in benzene and THF and stable as a brown suspension. CH_2Cl_2 dissolves **3** very well, but the CH_2Cl_2 solution of **3** gradually changes in color to dark brown along with formation of black insoluble materials and $[TiCl_2(tmeda)]_2(\mu\text{-}Cl)_2$ at room temperature. Thermolyzing **3** either in solid-state and in solution rapidly results in formation of dark brown products (no longer reddish).

In a J. Young valve NMR tube, complex **3** (10 mg) was suspended in THF-d_8 (ca. 0.6 mL). A ^{1}H NMR spectrum was collected at 25 °C as shown in Fig. S1. The NMR tube was heated at 50 °C in the NMR probe. The ^{1}H NMR spectrum after 30 min at 50 °C (Fig. S6) showed decomposition of **3** along with a complicated mixture of paramagnetic and diamagnetic species. A trace amount of ethylene was also formed, but methane was observed as a relatively major decomposition product. Deuterated methane CH_2D_2 or CH_3D was not observed. A GC-MS analysis of this decomposed sample after quenching with H_2O showed a complicated mixture probably by degradation of TMEDA.

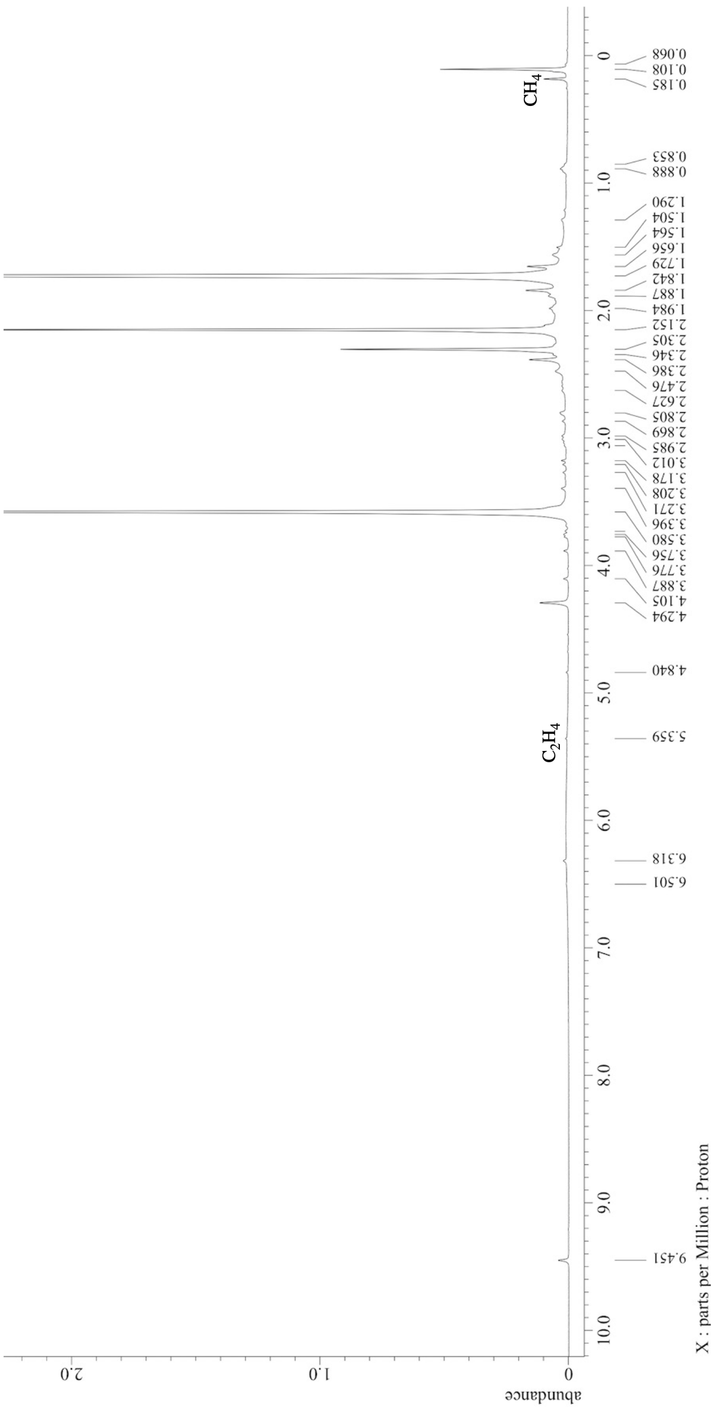

Fig. S6 ^{1}H NMR spectrum of decomposition of **3** at 50 °C for 30 min (400 MHz, in THF-d_8, at 25 °C).

Methylenation of Methyl Undecanoate by 3

Method A

To a reddish brown suspension of **3** (113 mg, 220 μmol) in THF (3 mL) was added methyl undecanoate (40.5 mg, 202 mmol) at room temperature. The reaction mixture was stirred for 18 hours, resulting in a gradual color change to yellowish brown. The reaction mixture was quenched by a saturated potassium carbonate solution (ca. 3 mL) under air and then filtered through a short plug of Celite. The organic phase was extracted with Et_2O (3 x 20 mL). The combined organic phase (colorless) was washed with brine, dried over sodium sulfate, filtered and evaporated by a rotary evaporator to give a pale yellow oil (36.1 mg). The molar ratio of 2-methoxy-1-decene (**4**) and 2-dodecanone (**5**) in the obtained mixture was determined by ^1H NMR spectroscopy with dibromomethane as an internal standard compared to the ^1H NMR spectrum in our previous paper[2] (**4**: 87.8 μmol, 43% yield; **5**: 66.7 μmol, 33% yield).

Method B

A solid mixture of **1a** (138 mg, 219 μmol) and **2** (153 mg, 447 μmol) was dissolved in THF (3 mL). Methyl undecanoate (38.7 mg, 193 mmol) was then added to the reddish brown mixture at room temperature. The reaction mixture was stirred for 18 hours, resulting in a gradual color change to yellowish brown. The reaction mixture was quenched by a saturated potassium carbonate solution (ca. 3 mL) under air and then filtered through a short plug of Celite. The organic phase was extracted with Et_2O (3 x 20 mL). The combined organic phase (colorless) was washed with brine, dried over sodium sulfate, filtered and evaporated by a rotary evaporator to give a pale yellow oil (35.6 mg). The molar ratio of 2-methoxy-1-decene and 2-dodecanone in the obtained mixture was determined by ^1H NMR spectroscopy with dibromomethane as an internal standard compared to the ^1H NMR spectrum in our previous paper[2] (**4**: 96.5 μmol, 50% yield; **5**: 48.3 μmol, 25% yield).

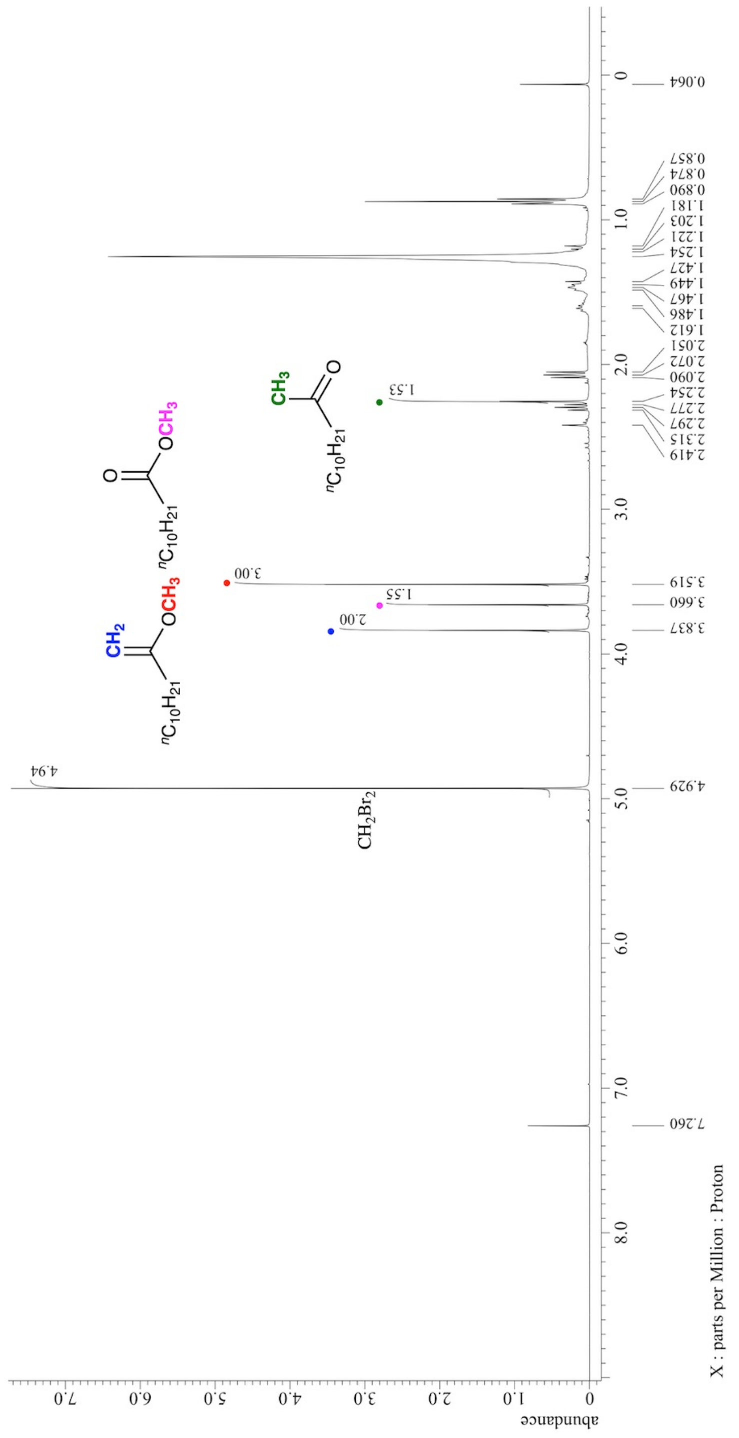

Fig. S7 ^{1}H NMR spectrum of resulting methylenation products by Method B with dibromomethane as an internal standard (400 MHz, in chloroform-d_1, at 25 °C).

Methylenation of γ-Undecanolactone by 3

To a reddish brown suspension of **3** (693 mg, 1.43 mmol) in THF (5 mL) was added γ-undecanolactone (187 mg, 1.01 mmol) at room temperature. The reaction mixture was stirred for 12 hours, resulting in a gradual color change to yellowish brown. The reaction mixture was quenched by a saturated potassium carbonate solution (ca. 5 mL) under air and then filtered through a short plug of Celite. The organic phase was extracted with Et_2O (3 x 15 mL). The combined organic phase (colorless) was washed with brine, dried over sodium sulfate, filtered and evaporated by a rotary evaporator to give a colorless oil (214 mg). The yield of the methylenation product, 2-heptyltetrahydro-5-methylenefuran,[9] in the obtained mixture was determined by ^{1}H NMR spectroscopy with 1,1,2,2-tetrachloroethane as an internal standard (353 μmol, 35% yield). The starting material γ-undecanolactone was consumed completely and some unidentified ring-opening and oligomerization products were observed.

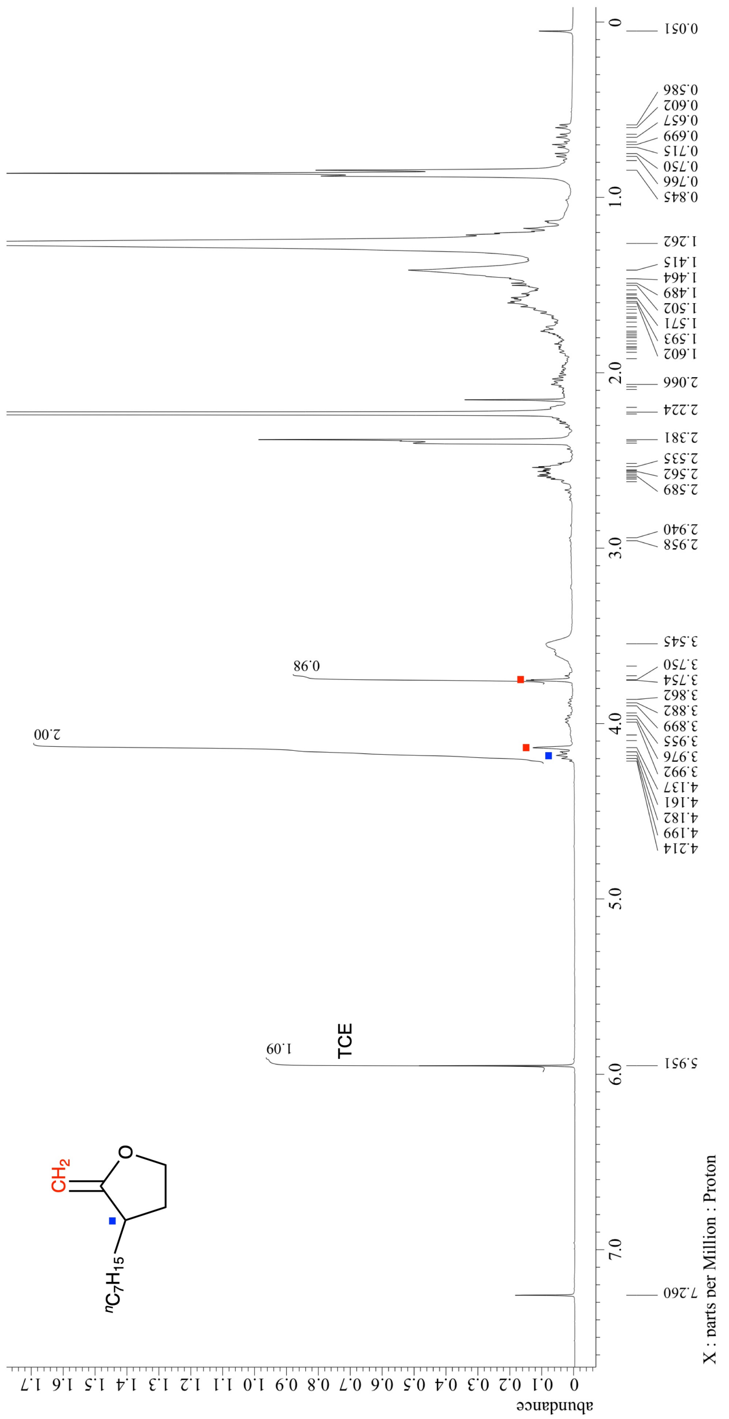

Fig. S8 ^{1}H NMR spectrum of resulting methylenation products with tetrachloroethane as an internal standard (400 MHz, in chloroform-d_1, at 25 °C).

Reaction of 4-Phenyl-1-butene with 3

To a reddish brown solution of **3** (970 mg, 2.21 mmol) in CH_2Cl_2 (40 mL) was added 6-phenyl-1-butene (245 mg, 1.85 mmol) at room temperature. The reaction mixture was heated at 60 °C and stirred for 18 hours, resulting in a gradual change to a dark brown suspension. The reaction mixture was quenched by diluted HCl aq (1 M, ca. 5 mL). The organic phase was separated and further extracted from the aqueous phase with Et_2O (5 x 5 mL). The combined organic phase was washed by brine (ca. 40 mL), dried by magnesium sulfate, filtered and evaporated by a rotary evaporator to give a pale yellow oil. The yields of olefin-metathesis and homologation products were determined by ^1H NMR and FID-GC analyses with mesitylene as an internal standard. **7**: 318 μmol, 34% yield (E : Z = 8 : 9); **8**: 70.0 μmol, 4% yield; **9**: 137 μmol, 7% yield, (E : Z = 6 : 1); **10**: 185 μmol, 10% yield; 6-phenyl-1-butene: 788 μmol, 43% recovery. The ^1H NMR spectra and GC retention times were compared to those of the independently synthesized[4-7] or commercially available samples. In addition, GC-MS analysis (Fig. S11) revealed further homologation products of **8**, **9**, and **10** ($[M]^+$: m/z = 160) and homologation of **7** ($[M]^+$: m/z = 250).

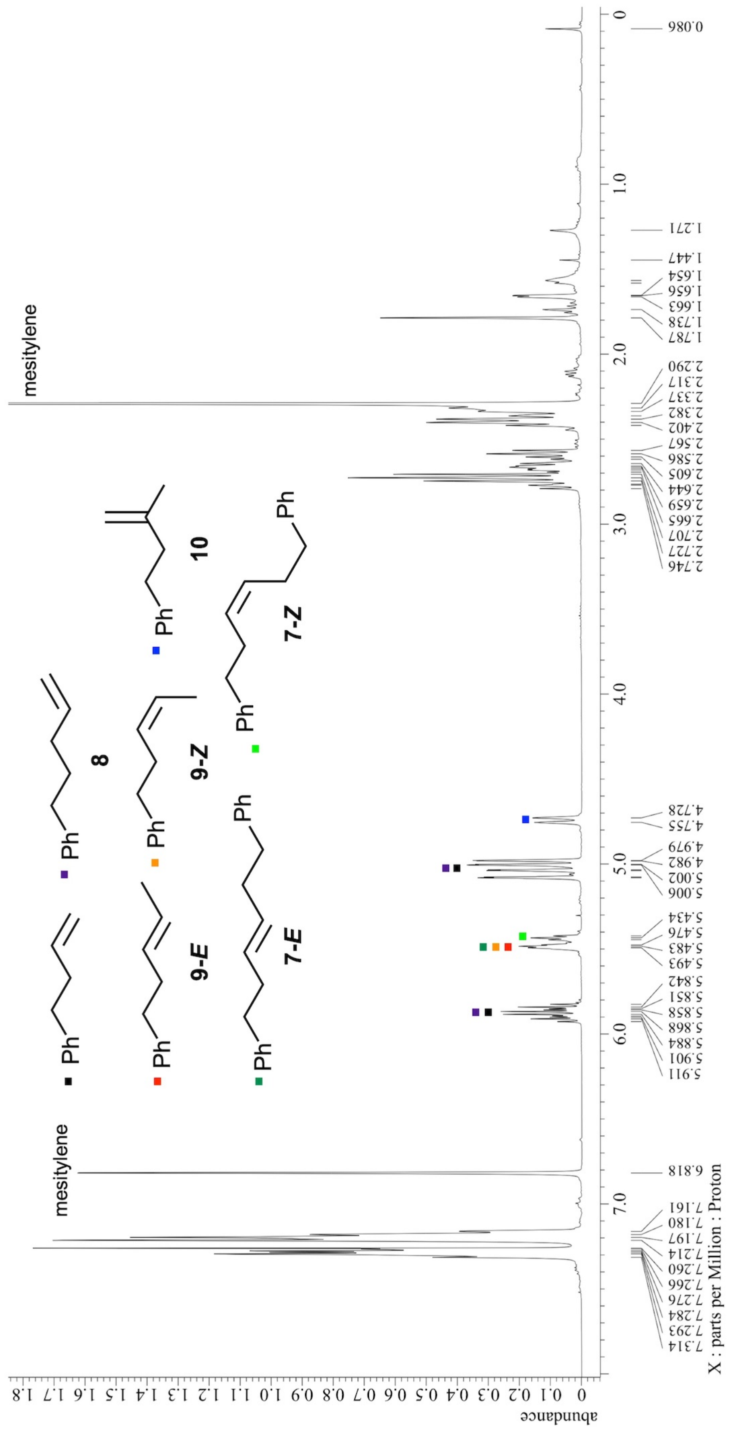

Fig. S9 ¹H NMR spectrum of the obtained oil from the reaction of **1** and 6-phenyl-1-butene (400 MHz, in chloroform-d_1, at 25 °C).

Cyclopropanation of 6-Phenylhexa-1,3-diene by 3

To a reddish brown solution of **3** (492 mg, 1.12 mmol) in CH_2Cl_2 (8 mL) was added 6-phenylhexa-1,3-diene (77.6 mg, 490 μmol) at room temperature. The reaction mixture was stirred at 40 °C for 6 hours, resulting in a gradual change to a dark brown suspension. The reaction mixture was quenched by diluted HCl aq (1 M, ca. 1.5 mL). The organic phase was extracted by CH_2Cl_2 (ca. 50 mL). The combined organic phase was washed with brine (ca. 15 mL), dried by magnesium sulfate, filtered and evaporated by a rotary evaporator to give a pale yellow oil (62.5 mg). Separation by column chromatography with a hexane eluent (R_f = 0.44) gave a mixture of 6-phenyl-hexa-1,3-diene and (*E*)-(4-cyclopropylbut-3-en-1-yl)benzene (**11-*E***) (392 μmol, 80% yield). The molar ratio of 6-phenylhexa-1,3-diene and (*E*)-(4-cyclopropylbut-3-en-1-yl)benzene in the obtained mixture was determined by ^1H NMR spectroscopy with ferrocene and FID-GC with mesitylene. An analytically pure sample for NMR and mass analyses was obtained by GPC separation with toluene. Formation of **11-*E*** was confirmed by comparison of the ^1H spectrum to the reported spectrum[10] and high-resolution mass spectroscopy. HRMS (FAB^+) Calcd for $C_{13}H_{16}$ $[M]^+$: 172.1252; $[M+H]^+$: 173.1330, Found: 173.1325. From the reaction mixture before quenching, brown crystals of $[TiCl(tmeda)]_2(\mu\text{-}Cl)_3$ were grown along with precipitation of greenish solid $[TiCl_2(tmeda)]_2(\mu\text{-}Cl)_2$ and black insoluble materials.

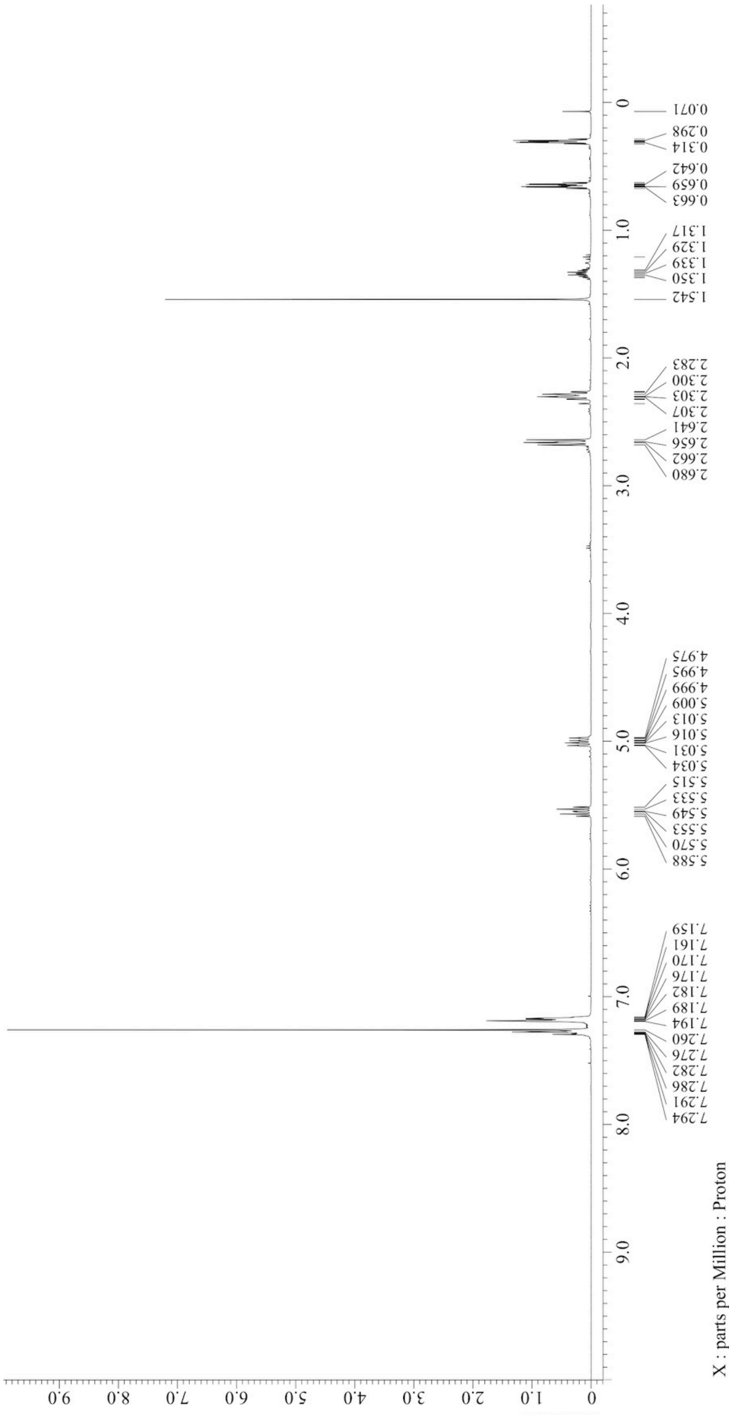

Fig. S10 ^{1}H NMR spectrum of (*E*)-(4-cyclopropylbut-3-en-1-yl)benzene (**11-*E***) after GPC separation (400 MHz, in chloroform-d_1, at 25 °C).

FID-GC and GC-MS Analyses

For FID-GC analyses, the solutions were transferred into a GC vial and injected into a Shimadzu GC-17A system. Separation was performed with nitrogen (3.0 mL/min, 55 cm/sec) as the carrier gas through a Zebron ZB-5MSplus GC column (30 m x 0.25 mm x 0.25 μm) with holding at 50 °C for 5 minutes, heating up to 300 °C with 10 °C/min rate, and then holding at 300 °C for 20 minutes.

For GC-MS analyses, the solutions were transferred into a GC vial and injected into a Shimadzu GC-2010 system equipped with a Shimadzu GCMS-QP2010 detector. Separation was performed with helium (2.0 mL/min, 52 cm/sec) as the carrier gas through a Zebron ZB-5MSplus GC column (30 m x 0.25 mm x 0.25 μm) with holding at 50 °C for 8 minutes, heating up to 300 °C with 10 °C/min rate, and then holding at 300 °C for 12 minutes.

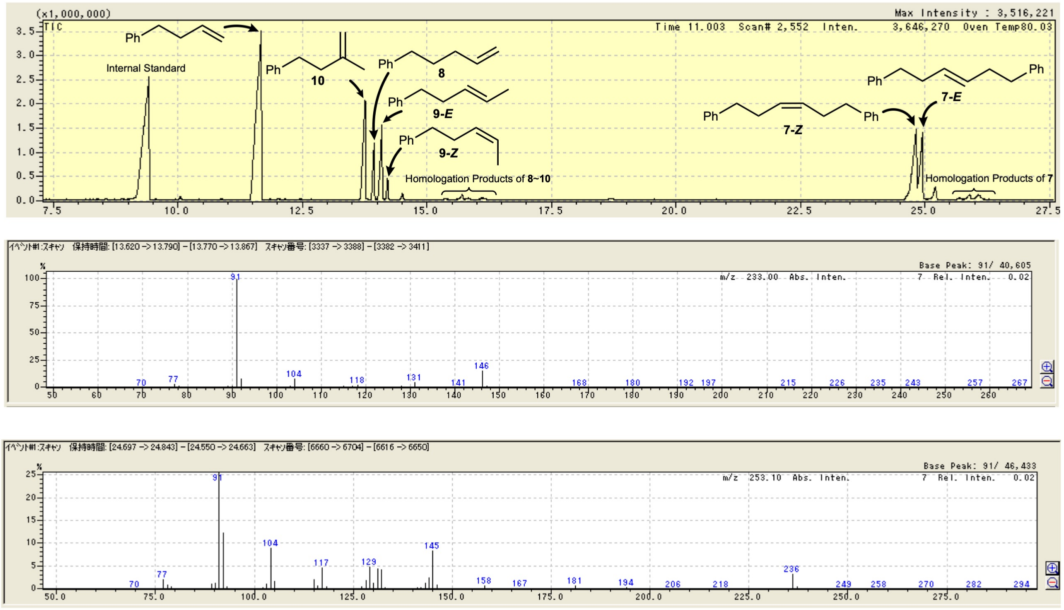

Fig. S11 GC-MS spectrum of reaction of 4-phenyl-1-butene with **3** in CH_2Cl_2 at 60 °C after 18 h (Top: Gas Chromatogram, Middle: Mass Spectrum around 13.7 min (**10**), Bottom: Mass Spectrum around 24.7 min (**7-*Z***)).

1,3-diene + **3** (1 equiv) at 25 °C

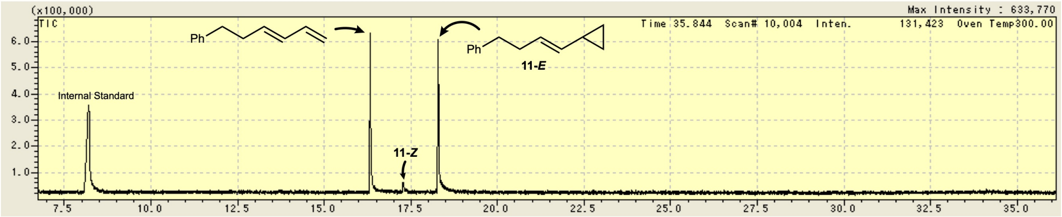

1,3-diene + **3** (1 equiv) at 80 °C

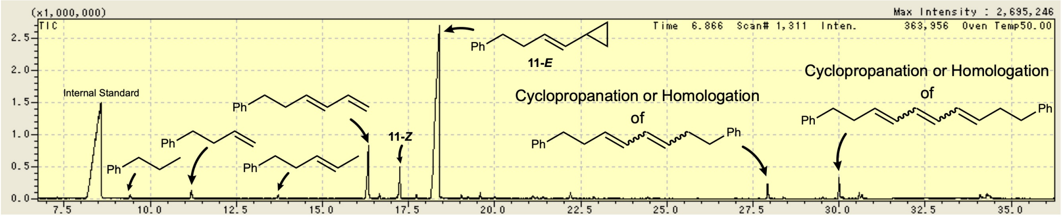

Fig. S12 GC-MS spectra of reaction of 6-phenyl-hexa-1,3-diene with **3** (1 equiv) in CH_2Cl_2 at 25 °C and 80 °C (Top: Clean formation of **11** at 25 °C, Bottom: Complicated mixture formed at 80 °C).

X-ray Crystallography

Crystallographic data are summarized Table S1-S6. Suitable crystals for X-ray analysis were placed on the end of a micro-mount coated with NVH oil. The X-ray intensity data collection was carried out on a Rigaku Varimax with a Saturn 944+ CCD area detector using graphite-monochromated Mo-Kα radiation (λ = 0.71075 Å) at 100(2) K. Preliminary indexing was performed from a set of twelve frames. Equivalent reflections were merged, and the collected images were processed by a Rigaku CrystalClear program. The initial structures were determined by the direct or Patterson method on SHELXS.[11] The further structure determination was performed by Fourier transform method and refined by least squares method on SHELXL.[11,12] All reflections were used during refinement with the exception of affected reflections by the beam-stopper. Non-hydrogen atoms were refined anisotropically and hydrogen atoms were refined using riding models, except for the methylene hydrogens in **1a** and **3**. For **1a**, the methylene hydrogens H1A and H1B were located from the difference map and refined isotropically. For **2**, the TMEDA and THF molecules are disordered over two positions. The thermal ellipsoids of these disorders were fixed by SHELXL restraints. For $[TiCl_2(tmeda)]_2(\mu\text{-}Cl)_2$, two crystallographically independent but chemically equivalent fragments of half $[TiCl_2(tmeda)]_2(\mu\text{-}Cl)_2$ molecules are present in the asymmetric unit. One TMEDA molecule was disordered over two positions. For **3**, the methylene hydrogen H39 was located from the difference map and refined isotropically. Some residual Q-peaks (>1) still remain due to the poor quality of the obtained crystal, but those Q-peaks are on unrealistic positions to locate any atom.

These results were checked using the IUCR's CheckCIF routine. The alerts in the output are related to the disordered groups.

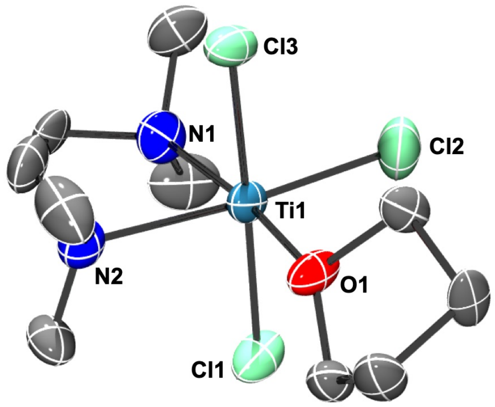

Fig. S13 POV-ray drawing of **2** with thermal ellipsoids at 50% probability level. Hydrogen atoms have been omitted for clarity.

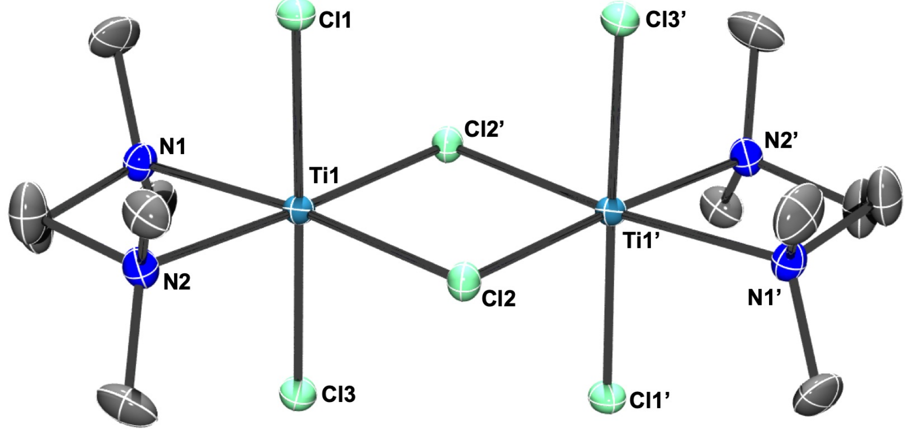

Fig. S14 POV-ray drawing of $[TiCl_2(tmeda)]_2(\mu\text{-}Cl)_2$ with thermal ellipsoids at 50% probability level. Hydrogen atoms and co-crystallized CH_2Cl_2 molecules have been omitted for clarity.

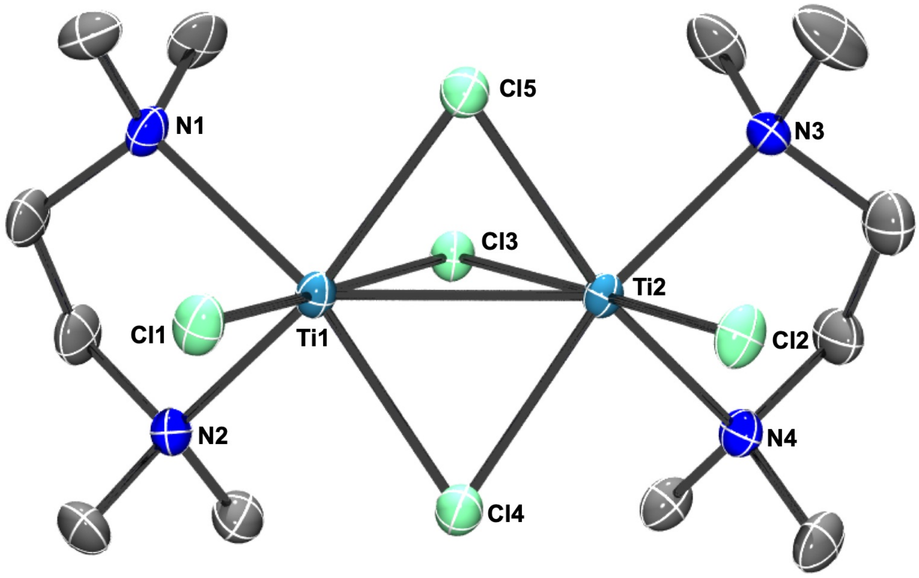

Fig. S15 POV-ray drawing of $[TiCl(tmeda)]_2(\mu\text{-}Cl)_3$ with thermal ellipsoids at 50% probability level. Hydrogen atoms have been omitted for clarity.

Table S1. Summary of Structure Determination of 1a

Empirical formula	$C_{13}H_{34}N_4I_2Zn_2$
Formula weight	630.98
Temperature	100(2) K
Wavelength	0.71075 Å
Crystal system	*Orthorhombic*
Space group	$P2_12_12_1$ (No. 19)
Cell constants:	
a	11.494(2) Å
b	13.090(2) Å
c	14.921(3) Å
α	90°
β	90°
γ	90°
Volume	2245.0(7) Å^3
Z	4
Density (calculated)	1.867 Mg/m^3
Absorption coefficient	4.887 mm^{-1}
F(000)	1224
Crystal size	0.08 x 0.06 x 0.03 mm^3
Theta range for data collection	2.730 to 27.480°
Index ranges	$-8 \leq h \leq 14, -16 \leq k \leq 16, -19 \leq l \leq 19$
Reflections collected	12448
Independent reflections	5031 [R (int) = 0.0644]
Completeness to theta = 27.55°	97.8 %
Absorption correction	Semi-empirical from equivalents
Max. and min. transmission	1.000 and 0.768
Refinement method	Full-matrix least-squares on F^2
Data / restraints / parameters	5031 / 0 /206
Goodness-of-fit on F^2	1.008
Final R indices [I>2sigma(I)]	$R_1 = 0.0315$, $wR_2 = 0.0581$
R indices (all data)	$R_1 = 0.0415$, $wR_2 = 0.0630$
Largest diff. peak and hole	0.770 and –0.853 e·Å^{-3}
Flack Parameter	0.011(19)

Table S2. Summary of Structure Determination of ZnClI(tmeda)

Empirical formula	$C_6H_{16}N_2ClIZn$
Formula weight	343.93
Temperature	100(2) K
Wavelength	0.71075 Å
Crystal system	*Monoclinic*
Space group	*C*2/*c* (No. 15)
Cell constants:	
a	23.149(11) Å
b	7.256(3) Å
c	15.032(7) Å
α	90°
β	111.169(6)°
γ	90°
Volume	2354.5(18) Å^3
Z	8
Density (calculated)	1.940 Mg/m^3
Absorption coefficient	4.888 mm^{-1}
F(000)	1328
Crystal size	0.14 x 0.12 x 0.08 mm^3
Theta range for data collection	2.837 to 27.511°
Index ranges	$-30 \leq h \leq 28, -9 \leq k \leq 9, -19 \leq l \leq 19$
Reflections collected	11250
Independent reflections	2707 [R (int) = 0.0294]
Completeness to theta = 27.55°	99.8 %
Absorption correction	Semi-empirical from equivalents
Max. and min. transmission	1.000 and 0.811
Refinement method	Full-matrix least-squares on F^2
Data / restraints / parameters	2707 / 0 /102
Goodness-of-fit on F^2	1.039
Final R indices [I>2sigma(I)]	R_1 = 0.0227, wR_2 = 0.0602
R indices (all data)	R_1 = 0.0263, wR_2 = 0.0613
Largest diff. peak and hole	0.0843 and –1.159 e·Å^{-3}

Table S3. Summary of Structure Determination of 2

Empirical formula	$C_{10}H_{24}N_2OCl_3Ti$
Formula weight	342.56
Temperature	100(2) K
Wavelength	0.71075 Å
Crystal system	*Monoclinic*
Space group	$P2_1/n$ (No. 14)
Cell constants:	
a	10.972(4) Å
b	12.308(4) Å
c	12.075(4) Å
α	90°
β	101.587(6)°
γ	90°
Volume	1597.4(9) Å^3
Z	4
Density (calculated)	1.424 Mg/m^3
Absorption coefficient	1.026 mm^{-1}
F(000)	726
Crystal size	0.12 x 0.10 x 0.07 mm^3
Theta range for data collection	3.257 to 27.458°
Index ranges	$-14 \le h \le 14, -15 \le k \le 15, -15 \le l \le 15$
Reflections collected	18771
Independent reflections	3644 [R (int) = 0.0284]
Completeness to theta = 27.55°	99.7 %
Absorption correction	Semi-empirical from equivalents
Max. and min. transmission	1.000 and 0.894
Refinement method	Full-matrix least-squares on F^2
Data / restraints / parameters	3644 / 60 /230
Goodness-of-fit on F^2	1.083
Final R indices [I>2sigma(I)]	R_1 = 0.0550, wR_2 = 0.1347
R indices (all data)	R_1 = 0.0693, wR_2 = 0.1447
Largest diff. peak and hole	0.620 and –0.806 e·Å^{-3}

Table S4. Summary of Structure Determination of $[TiCl_2(tmeda)]_2(\mu\text{-}Cl)_2\cdot 2CH_2Cl_2$

Empirical formula	$C_{14}H_{36}N_4Cl_{10}Ti_2$
Formula weight	710.77
Temperature	100(2) K
Wavelength	0.71075 Å
Crystal system	*Monoclinic*
Space group	$P2_1/c$ (No. 14)
Cell constants:	
a	13.348(3) Å
b	13.855(3) Å
c	17.166(4) Å
α	90°
β	112.468(4)°
γ	90°
Volume	2933.6(11) Å^3
Z	4
Density (calculated)	1.609 Mg/m^3
Absorption coefficient	1.467 mm^{-1}
F(000)	1448
Crystal size	0.10 x 0.08 x 0.07 mm^3
Theta range for data collection	2.941 to 27.484°
Index ranges	$-17 \leq h \leq 17, -17 \leq k \leq 17, -22 \leq l \leq 22$
Reflections collected	45330
Independent reflections	6710 [R (int) = 0.0433]
Completeness to theta = 27.55°	99.8 %
Absorption correction	Semi-empirical from equivalents
Max. and min. transmission	1.000 and 0.881
Refinement method	Full-matrix least-squares on F^2
Data / restraints / parameters	6710 / 0 / 314
Goodness-of-fit on F^2	1.158
Final R indices [I>2sigma(I)]	$R_1 = 0.0562$, $wR_2 = 0.1053$
R indices (all data)	$R_1 = 0.0622$, $wR_2 = 0.1084$
Largest diff. peak and hole	1.060 and –0.610 e·Å^{-3}

Table S5. Summary of Structure Determination of 3

Empirical formula	$C_{13}H_{34}N_4Cl_4Ti_2$
Formula weight	484.04
Temperature	100(2) K
Wavelength	0.71075 Å
Crystal system	*Monoclinic*
Space group	*C*2/*c* (No. 15)
Cell constants:	
a	23.348(11) Å
b	8.843(4) Å
c	12.648(6) Å
α	90°
β	121.198(6)°
γ	90°
Volume	2233.7(18) Å^3
Z	4
Density (calculated)	1.439 Mg/m^3
Absorption coefficient	1.196 mm^{-1}
F(000)	1008
Crystal size	0.10 x 0.08 x 0.01 mm^3
Theta range for data collection	3.223 to 27.465°
Index ranges	$-30 \le h \le 30$, $-11 \le k \le 8$, $-16 \le l \le 16$
Reflections collected	9148
Independent reflections	2548 [*R* (int) = 0.0367]
Completeness to theta = 27.55°	99.6 %
Absorption correction	Semi-empirical from equivalents
Max. and min. transmission	1.000 and 0.833
Refinement method	Full-matrix least-squares on F^2
Data / restraints / parameters	2548/ 0 / 113
Goodness-of-fit on F^2	1.055
Final R indices [I>2sigma(I)]	$R_1 = 0.0768$, $wR_2 = 0.1988$
R indices (all data)	$R_1 = 0.0851$, $wR_2 = 0.2057$
Largest diff. peak and hole	4.149 and –1.211 e·Å^{-3}

Table S5. Summary of Structure Determination of [$TiCl_2$(tmeda)]$_2$(μ-Cl)$_3$

Empirical formula	$C_{12}H_{32}N_4Cl_5Ti_2$
Formula weight	505.46
Temperature	100(2) K
Wavelength	0.71075 Å
Crystal system	*Orthorhombic*
Space group	$Pna2_1$ (No. 33)
Cell constants:	
a	19.796(5) Å
b	8.413(2) Å
c	13.102(3) Å
α	90°
β	90°
γ	90°
Volume	2182.1(9) $Å^3$
Z	4
Density (calculated)	1.539 Mg/m^3
Absorption coefficient	1.347 mm^{-1}
F(000)	1044
Crystal size	0.09 x 0.04 x 0.02 mm^3
Theta range for data collection	3.056 to 27.484°
Index ranges	$-25 \le h \le 16$, $-10 \le k \le 10$, $-17 \le l \le 17$
Reflections collected	20125
Independent reflections	4976 [*R* (int) = 0.0586]
Completeness to theta = 27.55°	99.8 %
Absorption correction	Semi-empirical from equivalents
Max. and min. transmission	1.000 and 0.855
Refinement method	Full-matrix least-squares on F^2
Data / restraints / parameters	2707 / 1 /216
Goodness-of-fit on F^2	1.096
Final R indices [I>2sigma(I)]	R_1 = 0.0507, wR_2 = 0.1171
R indices (all data)	R_1 = 0.0559, wR_2 = 0.1223
Largest diff. peak and hole	0.808 and –0.522 e·$Å^{-3}$
Flack Parameter	0.03(2)

Computational Details

Density functional calculations were carried out using the Gaussian09 program.[13] The model structure of **3** was optimized from the C_2 symmetric structure based on the experimental solid-state geometry. Geometrical optimization of the other structures were performed from model structures built on GaussView. The optimized structures were considered true minima if no imaginary vibration mode was obtained. Transition states were located to have a single imaginary vibration mode, which is corresponding to the reaction process. To reduce the computational cost, DFT methods were performed at B3LYP level using 6-31G(d,p) basis set for H, C and N and LanL2DZ basis for Cl and Ti for geometrical optimization. The reported values of free energy were reevaluated by additional single point calculations on each optimized geometry using B3LYP and cc-pVTZ for all atoms. Solvation calculations were performed at 298.150 K using the dielectric constant of $\varepsilon = 8.93$ for CH_2Cl_2.

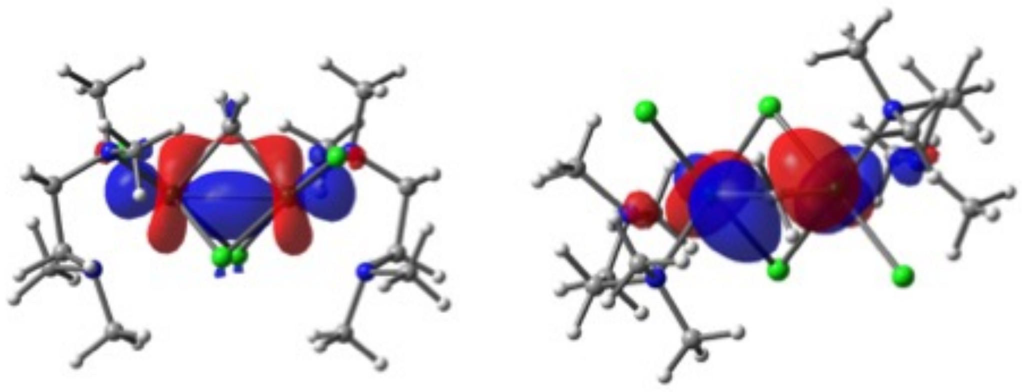

Fig. S16 HOMO (left) and HOMO-1 (right) in ^{1}**3** with 0.04 isovalue displaying a Ti–Ti interaction and an orbital interaction of the bridging CH_2 ligand between two Ti centers, respectively.

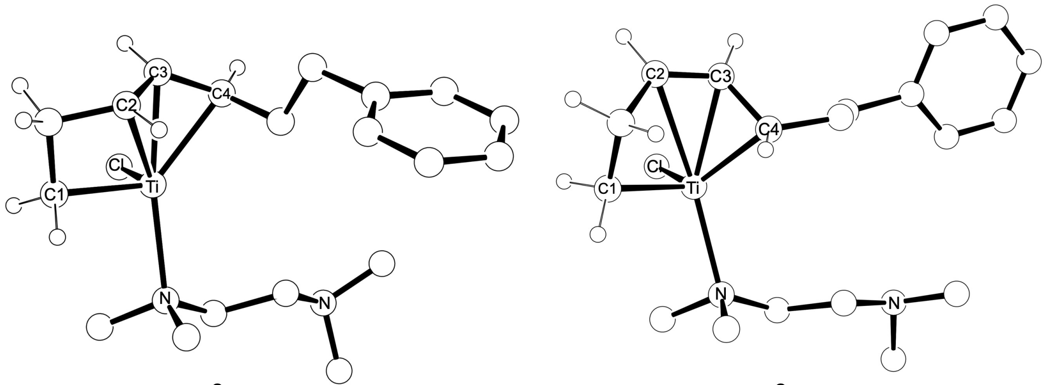

^{2}H’ **^{2}H’’**

Fig. S17 Optimized structures of two plausible *anti*-η^3-allyl intermediates **^{2}H’** (left) and **^{2}H’’** (right).

Table S6. Cartesian Coordinates of the Optimized Geometries

The cartesian coordinates of optimized geometries are given below in the standard XYZ format (units are in Å).

==============================

6-phenyl-hexa-1,3-diene

==============================

C	-3.428400	1.427600	-0.018400
C	-2.106300	1.189100	0.361600
C	-1.591700	-0.114500	0.403000
C	-2.439800	-1.175300	0.053800
C	-3.762500	-0.942300	-0.326800
C	-4.261600	0.361700	-0.364600
H	-3.808600	2.445100	-0.039800
H	-1.465300	2.024100	0.634200
H	-2.060100	-2.193900	0.085700
H	-4.404200	-1.778900	-0.589000
H	-5.291300	0.545000	-0.657200
C	-0.146900	-0.367200	0.776200
H	0.196700	0.398200	1.481100
H	-0.059600	-1.332500	1.289200
C	0.797300	-0.368800	-0.454300
H	0.732800	0.599600	-0.963800
H	0.430100	-1.124300	-1.163500
C	2.221600	-0.668600	-0.090000
H	2.403900	-1.636000	0.381700
C	3.263000	0.157800	-0.286600
H	3.091900	1.128500	-0.754200
C	4.636700	-0.155500	0.082900
H	4.800600	-1.127000	0.549700
C	5.682500	0.661500	-0.113400
H	5.561300	1.638000	-0.576000
H	6.688500	0.380500	0.180900

==============================

¹3

==============================

Ti	0.000000	1.318700	0.198300
Cl	1.800600	2.654400	1.137000
C	0.000000	0.000000	1.768700
Ti	0.000000	-1.318700	0.198300
Cl	-1.800600	-2.654400	1.137000
H	0.818300	0.346500	2.409400
H	-0.818300	-0.346500	2.409400
Cl	1.718600	0.049100	-1.152800
N	-1.711900	2.524000	1.194800
N	-0.489600	3.049100	-1.481300
C	-1.819800	3.568600	-1.075200
H	-2.045700	4.510700	-1.597600
H	-2.572200	2.838100	-1.382700
C	0.516300	4.141900	-1.463800
H	1.489300	3.737200	-1.744700
H	0.612700	4.571600	-0.469600
H	0.232400	4.928100	-2.178800
C	-3.009000	1.796700	1.256000
H	-2.862500	0.838300	1.754000
H	-3.390700	1.607200	0.255000
H	-3.743300	2.389500	1.817700
C	-0.554300	2.547600	-2.876300
H	-0.800600	3.364000	-3.570300
H	-1.305900	1.765100	-2.958600
H	0.416300	2.130000	-3.151000
C	-1.336300	2.874100	2.593900
H	-2.091000	3.543400	3.028700
H	-0.362200	3.361500	2.600400
H	-1.278600	1.962600	3.188300
C	-1.885900	3.794600	0.427200
H	-2.842900	4.263900	0.692900
H	-1.094400	4.478500	0.740000
Cl	-1.718600	-0.049100	-1.152800
N	1.711900	-2.524000	1.194800
N	0.489600	-3.049100	-1.481300
C	1.819800	-3.568600	-1.075200
H	2.045700	-4.510700	-1.597600
H	2.572200	-2.838100	-1.382700
C	-0.516300	-4.141900	-1.463800
H	-1.489300	-3.737200	-1.744700
H	-0.612700	-4.571600	-0.469600
H	-0.232400	-4.928100	-2.178800
C	3.009000	-1.796700	1.256000
H	2.862500	-0.838300	1.754000
H	3.390700	-1.607200	0.255000
H	3.743300	-2.389500	1.817700
C	0.554300	-2.547600	-2.876300
H	0.800600	-3.364000	-3.570300
H	1.305900	-1.765100	-2.958600
H	-0.416300	-2.130000	-3.151000
C	1.336300	-2.874100	2.593900
H	2.091000	-3.543400	3.028700
H	0.362200	-3.361500	2.600400
H	1.278600	-1.962600	3.188300
C	1.885900	-3.794600	0.427200
H	2.842900	-4.263900	0.692900
H	1.094400	-4.478500	0.740000

==============================

³3

==============================

Ti	0.262700	1.590300	0.388300
Cl	2.103100	2.717200	1.432000
C	-0.000000	-0.000000	1.691700
Ti	-0.262700	-1.590300	0.388300
Cl	-2.103100	-2.717200	1.432000

H	0.903900	-0.144000	2.303300
H	-0.903900	0.144000	2.303300
Cl	1.679400	-0.196500	-0.855100
N	-1.172300	3.227900	1.040600
N	0.249100	2.997600	-1.624000
C	-0.856800	3.952300	-1.346400
H	-0.759000	4.851000	-1.973900
H	-1.792900	3.466400	-1.631600
C	1.541100	3.717000	-1.741800
H	2.337700	2.990700	-1.913000
H	1.771500	4.255200	-0.824400
H	1.511700	4.423900	-2.584000
C	-2.601000	2.809500	0.955400
H	-2.761800	1.950700	1.608000
H	-2.864200	2.518500	-0.058200
H	-3.246400	3.636200	1.279100
C	0.000000	2.314300	-2.915000
H	-0.028500	3.040700	-3.740200
H	-0.944000	1.773000	-2.872000
H	0.802000	1.597000	-3.096300
C	-0.935300	3.663100	2.447200
H	-1.631400	4.469100	2.713000
H	0.090600	4.007600	2.560300
H	-1.100300	2.815000	3.114200
C	-0.912800	4.378700	0.118400
H	-1.696100	5.138300	0.246700
H	0.034100	4.831200	0.421900
Cl	-1.679400	0.196500	-0.855100
N	1.172300	-3.227900	1.040600
N	-0.249100	-2.997600	-1.624000
C	0.856800	-3.952300	-1.346400
H	0.759000	-4.851000	-1.973900
H	1.792900	-3.466400	-1.631600
C	-1.541100	-3.717000	-1.741800
H	-2.337700	-2.990700	-1.913000
H	-1.771500	-4.255200	-0.824400
H	-1.511700	-4.423900	-2.584000
C	2.601000	-2.809500	0.955400
H	2.761800	-1.950700	1.608000
H	2.864200	-2.518500	-0.058200
H	3.246400	-3.636200	1.279100
C	-0.000000	-2.314300	-2.915000
H	0.028500	-3.040700	-3.740200
H	0.944000	-1.773000	-2.872000
H	-0.802000	-1.597000	-3.096300
C	0.935300	-3.663100	2.447200
H	1.631400	-4.469100	2.713000
H	-0.090600	-4.007600	2.560300
H	1.100300	-2.815000	3.114200
C	0.912800	-4.378700	0.118400
H	1.696100	-5.138300	0.246700
H	-0.034100	-4.831200	0.421900

==============================

11

==============================

C	-3.897000	1.449200	-0.156100
C	-2.582300	1.226000	0.258000
C	-2.085100	-0.075900	0.412100
C	-2.943600	-1.150900	0.138900
C	-4.258600	-0.933500	-0.275800
C	-4.740300	0.369100	-0.425000
H	-4.263400	2.466300	-0.264600
H	-1.933200	2.072400	0.469700
H	-2.578000	-2.168200	0.258300
H	-4.908200	-1.780800	-0.477500
H	-5.764300	0.540600	-0.743900
C	-0.649000	-0.316200	0.824500
H	-0.293200	0.517500	1.440500
H	-0.589700	-1.216900	1.447900
C	0.305800	-0.483900	-0.384500
H	0.271100	0.421600	-1.002400
H	-0.077200	-1.303600	-1.009300
C	1.722700	-0.773500	0.026900
H	1.876900	-1.685000	0.607700
C	2.778700	-0.000900	-0.254400
H	2.626000	0.912800	-0.833100
C	4.173100	-0.281600	0.150400
H	4.306000	-1.191900	0.730800
C	5.323000	0.085300	-0.774600
H	5.073600	0.565300	-1.716700
H	6.162900	-0.600600	-0.830500
C	5.113100	0.863500	0.492400
H	4.721400	1.873700	0.413100
H	5.807600	0.716500	1.313900

==============================

[TiCl$_2$(tmeda)]$_2$(μ-Cl)$_2$

==============================

Ti	-0.015300	-1.948400	-0.008800
Cl	-0.258600	-1.943200	2.340000
Cl	0.151600	-1.958900	-2.359300
Cl	-1.729400	0.025100	-0.153000
N	1.519900	-3.648500	0.094900
C	2.642000	-3.487500	-0.876300
H	3.191400	-2.576700	-0.633800
H	2.249100	-3.405600	-1.886900
H	3.319900	-4.347400	-0.809300
C	2.150300	-3.805900	1.439700
H	2.893000	-4.612200	1.401600
H	1.402600	-4.035000	2.194300
H	2.642000	-2.872300	1.714500
C	0.736200	-4.876600	-0.249000
H	0.638400	-4.910100	-1.335900
H	1.290800	-5.772700	0.057800
C	-0.639800	-4.875400	0.408300
H	-0.545700	-4.785600	1.492300
H	-1.148100	-5.825700	0.197900
C	-2.035400	-4.041600	-1.418300
H	-2.701800	-4.910100	-1.345700
H	-1.241500	-4.250700	-2.130000
H	-2.600300	-3.181800	-1.778700
C	-2.653400	-3.561300	0.835800

H	-3.233200	-2.698500	0.503100
H	-2.318200	-3.389600	1.855500
H	-3.286300	-4.456600	0.799600
N	-1.478200	-3.731000	-0.068100
Ti	0.015300	1.948400	0.008800
Cl	0.258600	1.943200	-2.340000
Cl	-0.151600	1.958900	2.359300
Cl	1.729400	-0.025100	0.153000
N	-1.519900	3.648500	-0.094900
C	-2.642000	3.487500	0.876300
H	-3.191400	2.576700	0.633800
H	-2.249100	3.405600	1.886900
H	-3.319900	4.347400	0.809300
C	-2.150300	3.805900	-1.439700
H	-2.893000	4.612200	-1.401600
H	-1.402600	4.035000	-2.194300
H	-2.642000	2.872300	-1.714500
C	-0.736200	4.876600	0.249000
H	-0.638400	4.910100	1.335900
H	-1.290800	5.772700	-0.057800
C	0.639800	4.875400	-0.408300
H	0.545700	4.785600	-1.492300
H	1.148100	5.825700	-0.197900
C	2.035400	4.041600	1.418300
H	2.701800	4.910100	1.345700
H	1.241500	4.250700	2.130000
H	2.600300	3.181800	1.778700
C	2.653400	3.561300	-0.835800
H	3.233200	2.698500	-0.503100
H	2.318200	3.389600	-1.855500
H	3.286300	4.456600	-0.799600
N	1.478200	3.731000	0.068100

==============================

^{5}13

==============================

Ti	0.126500	-1.853000	-0.600400
Cl	-1.658100	0.107500	-0.616400
Cl	0.567600	-2.116900	-3.052700
N	1.601200	-3.586200	-0.069600
C	2.833500	-3.594400	-0.893300
H	3.402900	-2.684800	-0.690000
H	2.563500	-3.609400	-1.948800
H	3.459000	-4.466900	-0.654100
C	2.003300	-3.546200	1.356700
H	2.576900	-4.444300	1.628800
H	1.129900	-3.476500	2.007200
H	2.617200	-2.661500	1.527100
C	0.806800	-4.807900	-0.363400
H	0.708400	-4.875600	-1.450400
H	1.337700	-5.709800	-0.020700
C	-0.570100	-4.756700	0.288300
H	-0.471700	-4.709000	1.374900
H	-1.121000	-5.679400	0.061400
C	-2.095000	-3.872900	-1.404100
H	-2.828700	-4.670900	-1.224700
H	-1.403600	-4.181900	-2.188300
H	-2.619000	-2.976600	-1.742600
C	-2.318700	-3.170500	0.895300
H	-2.888600	-2.304700	0.554900
H	-1.783900	-2.900400	1.809500
H	-3.010700	-3.993700	1.119900
N	-1.346600	-3.563000	-0.156900
Ti	-0.126500	1.853000	0.600400
Cl	1.658100	-0.107500	0.616400
Cl	-0.567600	2.116900	3.052700
N	-1.601200	3.586200	0.069600
C	-2.833500	3.594400	0.893300
H	-3.402900	2.684800	0.690000
H	-2.563500	3.609400	1.948800
H	-3.459000	4.466900	0.654100
C	-2.003300	3.546200	-1.356700
H	-2.576900	4.444300	-1.628800
H	-1.129900	3.476500	-2.007200
H	-2.617200	2.661500	-1.527100
C	-0.806800	4.807900	0.363400
H	-0.708400	4.875600	1.450400
H	-1.337700	5.709800	0.020700
C	0.570100	4.756700	-0.288300
H	0.471700	4.709000	-1.374900
H	1.121000	5.679400	-0.061400
C	2.095000	3.872900	1.404100
H	2.828700	4.670900	1.224700
H	1.403600	4.181900	2.188300
H	2.619000	2.976600	1.742600
C	2.318700	3.170500	-0.895300
H	2.888600	2.304700	-0.554900
H	1.783900	2.900400	-1.809500
H	3.010700	3.993700	-1.119900
N	1.346600	3.563000	0.156900

==============================

^{2}A

==============================

Ti	0.652700	0.449200	0.493000
Cl	2.677200	1.081300	-0.709100
C	0.721500	0.450600	2.385100
H	1.645900	0.022600	2.810900
H	0.029800	0.764200	3.175600
N	-1.451000	0.933500	-0.221400
N	-0.052000	-1.631200	-0.126800
C	0.364800	-2.706800	0.804400
C	0.503900	-1.933800	-1.469700
C	-1.537700	-1.557400	-0.172900
C	-1.467500	2.075300	-1.170100
C	-2.242500	1.299300	0.982800
C	-2.016000	-0.286300	-0.868900
H	0.006700	-3.687700	0.457500
H	1.455000	-2.728300	0.860600
H	-0.029800	-2.496800	1.798200
H	0.109300	-2.885600	-1.852600
H	0.259400	-1.143000	-2.181000

H	1.590700	-1.999500	-1.399300
H	-1.956800	-2.435400	-0.686800
H	-1.895400	-1.582000	0.859600
H	-1.046500	2.956000	-0.679400
H	-0.862900	1.838100	-2.048300
H	-2.491500	2.305400	-1.494100
H	-1.783600	2.162700	1.467200
H	-3.274900	1.547900	0.701700
H	-2.251400	0.473100	1.693800
H	-3.114000	-0.252700	-0.866700
H	-1.700700	-0.285200	-1.915000

===============================

^{1}B

===============================

Ti	0.081300	0.050600	0.092000
Cl	-1.300100	-1.559900	1.102100
C	-0.603600	-0.132000	-1.916600
H	-1.028200	-1.117400	-2.094600
H	0.199400	0.140200	-2.597300
C	-1.394200	0.882300	-1.306300
H	-1.046400	1.905600	-1.455100
C	-2.838900	0.772800	-1.032900
H	-3.269000	1.564100	-0.416500
C	-3.675900	-0.175400	-1.486800
H	-3.287000	-0.969100	-2.125900
C	-5.139000	-0.257900	-1.154200
H	-5.736600	-0.257800	-2.077300
H	-5.447100	0.624200	-0.579400
C	-5.498600	-1.537700	-0.353200
H	-4.928700	-1.535000	0.582700
H	-5.166000	-2.413900	-0.922900
C	-6.978800	-1.650700	-0.060200
C	-7.848100	-2.265900	-0.973500
C	-7.523700	-1.111200	1.114300
C	-9.219900	-2.339600	-0.723000
H	-7.443900	-2.696300	-1.887000
C	-8.894800	-1.182200	1.369900
H	-6.864600	-0.636100	1.837500
C	-9.748700	-1.796900	0.450800
H	-9.874700	-2.824900	-1.441800
H	-9.295200	-0.761800	2.288500
H	-10.815000	-1.856300	0.649300
Cl	2.131000	-0.778700	1.885500
N	-0.664700	5.328700	-0.362400
N	-0.418400	2.001600	1.539900
C	-0.962000	3.264500	0.945300
H	-1.862200	2.996000	0.392000
H	-1.277100	3.921100	1.769900
C	-1.485100	1.487200	2.446200
H	-1.138100	0.588100	2.954200
H	-2.376600	1.239600	1.869200
H	-1.742500	2.246800	3.197800
C	-1.616000	5.125500	-1.453500
H	-2.108700	6.073600	-1.689800
H	-2.394400	4.414000	-1.165800
H	-1.134800	4.751700	-2.377500
C	0.754500	2.333300	2.393000
H	0.502600	3.150200	3.084400
H	1.601400	2.628100	1.775900
H	1.042400	1.454600	2.968300
C	0.321700	6.341000	-0.733300
H	0.943600	6.047100	-1.600000
H	0.988500	6.541200	0.111000
H	-0.190000	7.273800	-0.990700
C	-0.014500	4.080900	0.057200
H	0.336900	3.490900	-0.804500
H	0.874600	4.352400	0.632300
Ti	3.052700	-0.955800	-0.444300
Cl	3.893700	-0.816900	-2.634800
C	1.457900	-1.896600	-0.801000
H	1.345900	-2.089200	-1.874000
H	0.848400	-2.565900	-0.194400
Cl	2.134300	1.409200	-0.662400
N	4.205800	-2.876200	-0.036700
N	5.128500	-0.129400	0.610400
C	5.818700	-1.345000	1.111300
H	6.890800	-1.151500	1.261000
H	5.398300	-1.589500	2.089400
C	5.951500	0.541700	-0.431100
H	5.398700	1.394000	-0.829500
H	6.168300	-0.135400	-1.254400
H	6.895500	0.897800	0.004700
C	3.704800	-3.598900	1.170400
H	2.661400	-3.870600	1.014500
H	3.765700	-2.970500	2.055300
H	4.299500	-4.508100	1.324300
C	4.958000	0.839900	1.720300
H	5.936400	1.147400	2.114600
H	4.365200	0.393300	2.516400
H	4.431600	1.719700	1.346100
C	4.113200	-3.834600	-1.178100
H	4.722200	-4.722100	-0.965800
H	4.465800	-3.357700	-2.090700
H	3.072800	-4.132100	-1.309700
C	5.646600	-2.513300	0.152800
H	6.199600	-3.385800	0.525100
H	6.051400	-2.265800	-0.831000

===============================

^{1}B-TS

===============================

Ti	0.416100	0.067900	0.167000
Cl	-1.146500	-1.321400	1.352400
Ti	2.824100	-0.823400	-0.422200
Cl	3.633900	-0.822900	-2.710400
C	0.910600	-1.477600	-1.127800
H	1.263300	-1.789700	-2.108900
H	0.198000	-2.229000	-0.777400
Cl	2.143100	-0.517100	1.992200
N	-0.941900	5.308200	-0.410800
N	-0.124300	2.197900	1.684200

C	-0.863200	3.346200	1.077700
H	-1.762000	2.942300	0.609500
H	-1.201800	4.010200	1.888800
C	-1.003700	1.653100	2.749900
H	-0.515900	0.807700	3.234800
H	-1.941400	1.304300	2.316900
H	-1.219200	2.426000	3.503300
C	-1.865600	4.869500	-1.454800
H	-2.516600	5.701300	-1.741300
H	-2.506100	4.059700	-1.096300
H	-1.344600	4.515200	-2.365200
C	1.103300	2.705000	2.345800
H	0.862100	3.550900	3.007600
H	1.833600	3.022900	1.603700
H	1.546900	1.907000	2.939400
C	-0.144400	6.437400	-0.883700
H	0.502000	6.184200	-1.745400
H	0.494800	6.808500	-0.076800
H	-0.808500	7.251000	-1.192500
C	-0.089000	4.219100	0.080400
H	0.327400	3.618200	-0.744400
H	0.762900	4.676100	0.590600
C	-0.361600	-0.113200	-2.105700
H	-0.917000	-0.966200	-2.479000
H	0.430000	0.238900	-2.758000
C	-1.047500	0.845600	-1.311000
H	-0.662300	1.862700	-1.371600
C	-2.478300	0.722700	-0.965600
H	-2.832100	1.372500	-0.166500
C	-3.382900	-0.081500	-1.547200
H	-3.080600	-0.735300	-2.366400
C	-4.821400	-0.187500	-1.125600
H	-5.481100	-0.001200	-1.984700
H	-5.052400	0.578600	-0.375700
C	-5.166100	-1.585700	-0.546700
H	-4.522000	-1.770000	0.320000
H	-4.917300	-2.347700	-1.295600
C	-6.620200	-1.716300	-0.148700
C	-7.591100	-2.100900	-1.085600
C	-7.037000	-1.425500	1.158500
C	-8.937900	-2.192800	-0.728700
H	-7.286400	-2.337000	-2.102700
C	-8.382800	-1.515900	1.520800
H	-6.297800	-1.131900	1.900400
C	-9.338800	-1.900100	0.577400
H	-9.673000	-2.497800	-1.468500
H	-8.683400	-1.291300	2.540600
H	-10.385500	-1.974800	0.858200
Cl	2.248600	1.621300	-0.712900
N	3.436500	-3.004000	0.015000
N	5.054100	-0.527300	0.446900
C	5.350900	-1.823700	1.114400
H	6.426300	-1.907900	1.329200
H	4.826600	-1.834000	2.073100
C	6.045600	-0.273100	-0.634000
H	5.802700	0.667600	-1.128800
H	6.019600	-1.059600	-1.383600
H	7.055300	-0.205800	-0.205300
C	2.739600	-3.616900	1.181400
H	1.661200	-3.541000	1.038700
H	3.000000	-3.098300	2.101300
H	3.021700	-4.674000	1.268800
C	5.205100	0.580600	1.426000
H	6.240200	0.629900	1.791300
H	4.532200	0.432800	2.267900
H	4.956000	1.524000	0.937000
C	3.157800	-3.874600	-1.163500
H	3.580600	-4.874200	-0.998900
H	3.592200	-3.433600	-2.059600
H	2.079600	-3.967500	-1.296000
C	4.913400	-3.000800	0.255900
H	5.214900	-3.942400	0.733800
H	5.405600	-2.962800	-0.718100

================================

[1]C

================================

Ti	0.545000	-0.023100	0.472500
Cl	-0.734400	-1.022800	2.276300
C	0.175100	-2.353700	-0.549700
Ti	3.019700	-0.492800	-0.411100
Cl	3.048700	-1.465800	-2.579100
H	-0.528800	-2.900400	0.078300
H	0.430000	-2.980600	-1.411100
C	-0.449800	-1.038400	-1.063700
H	0.050600	-0.723800	-1.982900
C	1.478100	-2.056600	0.262600
H	2.279900	-2.648400	-0.185700
H	1.423200	-2.373700	1.304800
C	-1.916400	-0.909300	-1.193900
H	-2.246300	-0.099300	-1.846700
C	-2.868900	-1.687000	-0.645700
H	-2.581700	-2.500300	0.019800
C	-4.346200	-1.532400	-0.875300
H	-4.739700	-2.429400	-1.376500
H	-4.543200	-0.688800	-1.548200
C	-5.144700	-1.341800	0.440700
H	-4.808000	-0.419800	0.928000
H	-4.897500	-2.166000	1.121100
C	-6.641400	-1.296300	0.221200
C	-7.390000	-2.480700	0.143400
C	-7.312700	-0.076200	0.056200
C	-8.765600	-2.448400	-0.092900
H	-6.889200	-3.437100	0.275800
C	-8.688900	-0.037900	-0.180100
H	-6.752200	0.853600	0.120100
C	-9.420800	-1.225000	-0.256200
H	-9.326500	-3.377700	-0.144300
H	-9.189400	0.919100	-0.300500
H	-10.491700	-1.197500	-0.436500
Cl	2.796600	0.422900	1.908400
N	-2.926200	3.608300	-0.704100

N	-0.126000	2.174200	1.483000
C	-1.500000	2.567800	1.029800
H	-2.156900	1.727900	1.271000
H	-1.830400	3.431400	1.626200
C	-0.199700	2.042100	2.967300
H	0.755500	1.678200	3.345800
H	-0.978200	1.334800	3.240800
H	-0.418500	3.021100	3.415800
C	-4.052800	2.679200	-0.651900
H	-4.985700	3.230100	-0.807100
H	-4.112300	2.195200	0.326000
H	-3.991700	1.889100	-1.423700
C	0.834400	3.282200	1.229800
H	0.468600	4.210200	1.691600
H	0.981600	3.443400	0.166300
H	1.795400	3.018900	1.671900
C	-2.910300	4.313000	-1.983800
H	-2.766300	3.639200	-2.849900
H	-2.105300	5.054200	-1.992900
H	-3.859800	4.839100	-2.124300
C	-1.637100	2.956500	-0.449400
H	-1.488100	2.081200	-1.106600
H	-0.861200	3.679600	-0.710400
Cl	1.684700	1.428000	-1.304200
N	4.718900	-1.972200	0.376600
N	4.905000	0.889300	-0.535500
C	5.966900	0.196500	0.247700
H	6.950300	0.626100	0.010200
H	5.772900	0.391800	1.304900
C	5.294700	0.992500	-1.971000
H	4.499300	1.502800	-2.515300
H	5.429300	0.007400	-2.412000
H	6.225000	1.568000	-2.066200
C	4.705800	-2.200900	1.848400
H	3.747100	-2.633600	2.136800
H	4.831100	-1.265400	2.387900
H	5.512300	-2.892300	2.125700
C	4.772000	2.277700	-0.018900
H	5.726900	2.809600	-0.122700
H	4.480500	2.251700	1.029400
H	4.004100	2.800600	-0.588300
C	4.713000	-3.318900	-0.265600
H	5.626700	-3.864700	0.003100
H	4.651500	-3.215200	-1.347700
H	3.856800	-3.894400	0.089700
C	5.991600	-1.298000	-0.021400
H	6.836900	-1.751600	0.514300
H	6.147100	-1.490000	-1.085300

==============================

^{3}C

==============================

Ti	0.262300	0.500500	0.503700
Cl	-1.162700	-0.585400	2.138600
C	0.207600	-1.794200	-1.263000
Ti	3.207000	-0.760700	-0.426500
Cl	3.696500	-1.538000	-2.664900
H	-0.435200	-2.665500	-1.093100
H	0.618700	-1.895900	-2.273700
C	-0.619700	-0.503800	-1.173200
H	-0.261400	0.238600	-1.899100
C	1.365900	-1.821700	-0.240700
H	1.984100	-2.717400	-0.443700
H	0.987400	-1.976800	0.778200
C	-2.093100	-0.568700	-1.219600
H	-2.582500	0.374000	-1.471600
C	-2.897500	-1.624400	-0.991200
H	-2.467000	-2.589600	-0.724700
C	-4.398800	-1.574000	-1.037200
H	-4.781900	-2.297500	-1.771800
H	-4.733600	-0.583600	-1.369600
C	-5.048200	-1.898400	0.334700
H	-4.683600	-1.175000	1.072700
H	-4.701100	-2.885700	0.663300
C	-6.560400	-1.877100	0.289100
C	-7.286400	-3.022400	-0.071200
C	-7.272400	-0.701800	0.570100
C	-8.680300	-2.995100	-0.150300
H	-6.753000	-3.945600	-0.286200
C	-8.666400	-0.668900	0.493000
H	-6.727200	0.194400	0.857700
C	-9.376000	-1.816700	0.131400
H	-9.223000	-3.895000	-0.426800
H	-9.198200	0.251200	0.719800
H	-10.460500	-1.794500	0.074400
Cl	2.420400	0.248600	1.917700
N	-3.036100	4.080900	-0.916900
N	-0.234800	2.619200	1.257700
C	-1.583100	3.117200	0.823700
H	-2.301600	2.340700	1.098000
H	-1.824200	4.013500	1.411800
C	-0.261100	2.561700	2.752200
H	0.681300	2.149400	3.115000
H	-1.077300	1.921400	3.080800
H	-0.394400	3.572500	3.158100
C	-4.111600	3.092000	-0.951500
H	-5.071200	3.603200	-1.074200
H	-4.155400	2.528100	-0.016800
H	-4.003400	2.370600	-1.783700
C	0.830300	3.593900	0.886500
H	0.578700	4.590300	1.273600
H	0.957400	3.638600	-0.192400
H	1.772800	3.267500	1.327800
C	-3.032400	4.871100	-2.146200
H	-2.830500	4.266700	-3.050600
H	-2.273200	5.656500	-2.084400
H	-4.008000	5.349800	-2.275000
C	-1.719700	3.492200	-0.659000
H	-1.519400	2.627600	-1.319300
H	-0.975300	4.252800	-0.906700
Cl	2.093500	1.367000	-1.286800
N	4.613800	-2.325600	0.504300

N	5.170600	0.568200	-0.055700
C	6.056300	-0.291900	0.777700
H	7.096900	0.055600	0.711800
H	5.744500	-0.176500	1.818300
C	5.794000	0.823100	-1.384900
H	5.111900	1.428700	-1.983200
H	5.975000	-0.109000	-1.916500
H	6.741900	1.363200	-1.258000
C	4.301400	-2.569000	1.941300
H	3.292400	-2.976100	2.024300
H	4.339000	-1.643000	2.510000
H	5.014500	-3.289100	2.362800
C	5.005800	1.884200	0.612000
H	5.980900	2.378000	0.719800
H	4.553000	1.746300	1.592100
H	4.348600	2.508400	0.006400
C	4.592000	-3.648000	-0.184000
H	5.363200	-4.303900	0.239400
H	4.762600	-3.510200	-1.250200
H	3.618600	-4.119500	-0.036200
C	5.989000	-1.754400	0.361700
H	6.695800	-2.339800	0.965200
H	6.280700	-1.869500	-0.684700

==============================

^{1}D

==============================

Ti	0.222100	1.778000	-0.101300
Cl	-1.722900	2.386300	1.208600
C	0.145900	-0.401700	-2.533000
Ti	2.235700	-1.479700	-0.475400
Cl	3.821200	-2.175000	-2.157500
H	-0.518200	-0.758500	-3.340000
H	1.051400	-0.044700	-3.033500
C	-0.549300	0.794700	-1.867800
H	-0.422800	1.661700	-2.534600
C	0.508300	-1.612800	-1.653600
H	0.773100	-2.450200	-2.313200
H	-0.335000	-1.926600	-1.031100
C	-2.007100	0.635800	-1.603400
H	-2.549700	1.562100	-1.415500
C	-2.737600	-0.495700	-1.568300
H	-2.263900	-1.459600	-1.747200
C	-4.210000	-0.539200	-1.263000
H	-4.758200	-1.026300	-2.082900
H	-4.608300	0.479600	-1.180500
C	-4.521700	-1.311000	0.047000
H	-3.990800	-0.821100	0.870800
H	-4.112100	-2.325500	-0.037000
C	-6.001300	-1.381700	0.355100
C	-6.804000	-2.394800	-0.190800
C	-6.614000	-0.414900	1.165900
C	-8.176100	-2.441500	0.063800
H	-6.346400	-3.157400	-0.817200
C	-7.985900	-0.457100	1.424400
H	-6.007000	0.375000	1.602600
C	-8.772700	-1.471300	0.873400
H	-8.778300	-3.237700	-0.365500
H	-8.438900	0.299300	2.059600
H	-9.839300	-1.508200	1.076100
Cl	0.740300	-0.323600	1.271500
N	0.188300	3.864200	-1.167700
N	1.612300	3.315100	1.452100
C	1.799600	4.539700	0.639900
H	2.074100	5.393000	1.279300
H	2.639300	4.369400	-0.037800
C	0.873400	3.639100	2.697800
H	0.690700	2.719600	3.256100
H	-0.091000	4.088200	2.473800
H	1.463700	4.327800	3.320900
C	1.155500	3.924900	-2.297900
H	0.862500	3.209600	-3.067200
H	2.157600	3.663400	-1.963300
H	1.165800	4.931900	-2.736100
C	2.933200	2.793800	1.862600
H	3.479200	3.540500	2.458900
H	3.521300	2.528000	0.986000
H	2.783100	1.900700	2.469000
C	-1.157500	4.213200	-1.698000
H	-1.153700	5.230200	-2.112800
H	-1.889300	4.147400	-0.893200
H	-1.433900	3.516800	-2.491000
C	0.547500	4.895400	-0.146200
H	0.689400	5.865200	-0.643900
H	-0.307000	4.995100	0.526500
Cl	2.574000	1.025300	-0.945100
N	1.815700	-3.672800	0.212600
N	3.883100	-1.824400	1.385000
C	3.479800	-3.110700	2.003900
H	4.310400	-3.537800	2.585500
H	2.670000	-2.906900	2.708400
C	5.259900	-1.930600	0.835400
H	5.526300	-0.990700	0.348800
H	5.321100	-2.720500	0.089700
H	5.977200	-2.132100	1.644100
C	0.619800	-3.776400	1.092300
H	-0.266800	-3.486600	0.528300
H	0.708400	-3.108300	1.946800
H	0.496900	-4.809200	1.445900
C	3.903600	-0.767900	2.421300
H	4.602400	-1.028900	3.229400
H	2.904500	-0.631300	2.833100
H	4.228000	0.168600	1.966400
C	1.622600	-4.618400	-0.921600
H	1.571800	-5.650600	-0.549900
H	2.451700	-4.520800	-1.622400
H	0.690000	-4.387800	-1.436400
C	3.032100	-4.118000	0.957700
H	2.833700	-5.086000	1.438900
H	3.823300	-4.276200	0.221100

==============================

^{3}D

==============================

Ti	0.308000	1.810300	-0.082500
Cl	-1.622000	2.587200	1.186800
C	0.147800	-0.321600	-2.554600
Ti	2.163500	-1.511600	-0.459600
Cl	3.826500	-2.242500	-2.091600
H	-0.530800	-0.630800	-3.369100
H	1.057100	0.036200	-3.046500
C	-0.518000	0.868200	-1.843600
H	-0.377300	1.752900	-2.484000
C	0.492200	-1.577100	-1.730400
H	0.770800	-2.376200	-2.429800
H	-0.367100	-1.924900	-1.148600
C	-1.978200	0.742400	-1.571300
H	-2.497100	1.678600	-1.366600
C	-2.732600	-0.373400	-1.551200
H	-2.279400	-1.343600	-1.748200
C	-4.205500	-0.392800	-1.246700
H	-4.763200	-0.841800	-2.081800
H	-4.581300	0.631400	-1.132700
C	-4.535100	-1.199800	0.037300
H	-3.998200	-0.747000	0.878200
H	-4.143900	-2.218400	-0.077900
C	-6.016800	-1.253700	0.339000
C	-6.832400	-2.244800	-0.227600
C	-6.617700	-0.292600	1.165300
C	-8.205900	-2.275900	0.022300
H	-6.384100	-3.002700	-0.866300
C	-7.990800	-0.319300	1.418900
H	-6.000600	0.480200	1.618000
C	-8.790700	-1.311800	0.847400
H	-8.818300	-3.055500	-0.422900
H	-8.434700	0.432100	2.066400
H	-9.858400	-1.336700	1.046500
Cl	0.521400	-0.364100	1.274400
N	0.417000	3.899600	-1.156600
N	1.784800	3.219900	1.479300
C	2.082200	4.417200	0.656100
H	2.437500	5.242900	1.291600
H	2.901600	4.164300	-0.020900
C	1.059300	3.618600	2.712800
H	0.798100	2.723800	3.280000
H	0.135900	4.139700	2.472700
H	1.696600	4.265500	3.333800
C	1.371600	3.895900	-2.298700
H	1.025100	3.198300	-3.062300
H	2.360500	3.575000	-1.977700
H	1.436800	4.899200	-2.740000
C	3.057000	2.604300	1.916100
H	3.660100	3.323200	2.490400
H	3.623500	2.260100	1.052600
H	2.830700	1.750800	2.555400
C	-0.904200	4.353500	-1.669600
H	-0.825600	5.369100	-2.079800
H	-1.631200	4.338200	-0.858200
H	-1.241000	3.685200	-2.463800
C	0.867800	4.888600	-0.130100
H	1.099300	5.844000	-0.621900
H	0.025300	5.063800	0.541900
Cl	2.641300	0.986100	-0.984200
N	1.644900	-3.742500	0.081200
N	3.667900	-2.020500	1.494200
C	3.237000	-3.354500	1.979700
H	4.044800	-3.835100	2.551100
H	2.403800	-3.210800	2.671100
C	5.075900	-2.078700	1.023900
H	5.359900	-1.107100	0.615200
H	5.189400	-2.819400	0.235000
H	5.746400	-2.325800	1.859700
C	0.419200	-3.848400	0.916700
H	-0.445000	-3.545900	0.325200
H	0.477800	-3.190800	1.782200
H	0.274100	-4.884000	1.253300
C	3.614900	-1.056000	2.616000
H	4.236000	-1.401300	3.455200
H	2.585900	-0.927600	2.950200
H	3.997500	-0.093800	2.275000
C	1.462100	-4.622300	-1.105200
H	1.374600	-5.670600	-0.789200
H	2.314100	-4.509500	-1.775500
H	0.551500	-4.341200	-1.633500
C	2.825100	-4.263100	0.834400
H	2.598000	-5.265200	1.225500
H	3.642700	-4.367700	0.117400

==============================

^{2}E

==============================

Ti	2.561800	-0.540700	0.481700
C	1.851900	-0.358200	2.243200
H	2.319400	0.385500	2.908200
H	1.029100	-0.859300	2.764400
Cl	3.594100	-1.835500	-1.491800
N	4.543100	0.652400	0.695300
N	1.988000	1.419300	-0.649200
C	0.769500	2.083000	-0.122200
C	1.773100	1.126300	-2.089900
C	3.150400	2.344600	-0.494000
C	5.758100	-0.193100	0.569800
C	4.643900	1.388800	1.981300
C	4.482000	1.604300	-0.450300
H	0.572500	3.021700	-0.661800
H	-0.082400	1.413300	-0.242000
H	0.899900	2.287900	0.940300
H	1.581500	2.053800	-2.648100
H	2.641600	0.617700	-2.507700
H	0.909300	0.468400	-2.197500
H	3.166000	3.076100	-1.313600
H	3.006300	2.909600	0.429200
H	5.777900	-0.910600	1.393600
H	5.725300	-0.739900	-0.371400

H	6.665500	0.425200	0.615300
H	4.725400	0.669000	2.796100
H	5.531600	2.036500	1.981400
H	3.758800	1.998800	2.156300
H	5.303400	2.333400	-0.386200
H	4.627100	1.022400	-1.364300
C	-7.509400	-0.090600	1.400600
C	-6.314800	-0.663200	0.959100
C	-5.725000	-0.262000	-0.248600
C	-6.366100	0.730300	-1.004400
C	-7.561000	1.306300	-0.567300
C	-8.136800	0.897800	0.638100
H	-7.952000	-0.419300	2.336900
H	-5.834800	-1.435800	1.555500
H	-5.927300	1.049800	-1.947000
H	-8.044100	2.070400	-1.170200
H	-9.067900	1.342200	0.977900
C	-4.410400	-0.859000	-0.702400
H	-4.338600	-1.900400	-0.368400
H	-4.366700	-0.873300	-1.798000
C	-3.181500	-0.081200	-0.160900
H	-3.217600	-0.065300	0.934600
H	-3.263900	0.962400	-0.496600
C	-1.878400	-0.665400	-0.623500
H	-1.697800	-0.654300	-1.700100
C	-0.958400	-1.226700	0.185200
H	-1.141500	-1.256300	1.258900
C	0.266300	-1.860700	-0.281300
H	0.478000	-1.799800	-1.348400
C	1.070300	-2.647800	0.490600
H	1.854100	-3.244800	0.040100
H	0.840200	-2.827200	1.534500

==============================

^{2}E’

==============================

Ti	2.710600	0.668200	-0.068000
C	3.332500	1.578500	-1.628700
H	4.003500	1.051800	-2.323900
H	3.148000	2.597900	-1.989300
Cl	2.463800	0.612100	2.457400
N	4.295300	-1.045300	0.009000
N	1.399400	-1.145200	-0.700900
C	0.648200	-0.934500	-1.962900
C	0.421600	-1.441400	0.378900
C	2.327000	-2.300800	-0.867000
C	5.220200	-0.940900	1.166000
C	5.125100	-1.124800	-1.220000
C	3.463100	-2.273200	0.149700
H	0.082000	-1.837700	-2.235900
H	-0.050000	-0.107600	-1.828900
H	1.343200	-0.681300	-2.763500
H	-0.170600	-2.332500	0.126500
H	0.935900	-1.602600	1.326200
H	-0.247400	-0.588200	0.496500
H	1.776500	-3.247900	-0.776600
H	2.728700	-2.263300	-1.881900
H	5.817500	-0.032200	1.061900
H	4.645200	-0.876900	2.088700
H	5.894600	-1.808200	1.201700
H	5.738100	-0.226700	-1.293800
H	5.777400	-2.008800	-1.183100
H	4.501100	-1.182000	-2.110900
H	4.083500	-3.173900	0.031500
H	3.064600	-2.283600	1.167800
C	-6.484700	-1.398900	1.380600
C	-5.207500	-1.065300	0.925100
C	-5.033000	-0.318000	-0.248900
C	-6.174400	0.086400	-0.956400
C	-7.453800	-0.244700	-0.505200
C	-7.613100	-0.988800	0.666300
H	-6.598100	-1.982000	2.290400
H	-4.333700	-1.392000	1.484300
H	-6.058300	0.661500	-1.872200
H	-8.325000	0.074400	-1.070600
H	-8.607300	-1.250300	1.017300
C	-3.649000	0.074100	-0.719900
H	-2.928400	-0.705100	-0.446600
H	-3.637400	0.147800	-1.814100
C	-3.175500	1.424500	-0.122000
H	-3.161700	1.356300	0.971800
H	-3.921600	2.189500	-0.381000
C	-1.830500	1.852000	-0.631900
H	-1.742500	1.974500	-1.713300
C	-0.754700	2.109500	0.136600
H	-0.834800	2.005700	1.219200
C	0.518200	2.596700	-0.381600
H	0.611500	2.670500	-1.462500
C	1.528100	3.056600	0.402600
H	1.440300	3.057500	1.483500
H	2.397100	3.541100	-0.025300

==============================

^{2}F

==============================

Ti	1.639000	-0.714400	-0.410500
Cl	0.900300	1.321700	0.599100
C	2.086300	-0.655300	-2.218800
H	2.063400	0.090800	-3.018600
H	2.437600	-1.640000	-2.575200
N	5.681800	1.889100	-0.393900
N	3.619200	-1.159600	0.676500
C	4.018900	-2.578600	0.473300
C	3.437800	-0.936000	2.138200
C	4.748200	-0.322400	0.152600
C	5.309200	3.219600	-0.870600
C	6.810600	1.978600	0.530600
C	4.531300	1.193600	0.193400
H	4.990400	-2.774700	0.947300
H	3.276200	-3.241800	0.915900
H	4.099400	-2.788500	-0.595200
H	4.377600	-1.114100	2.678200

H	3.100200	0.082800	2.325800
H	2.680800	-1.627100	2.516100
H	5.651600	-0.592400	0.718500
H	4.902100	-0.612000	-0.888600
H	6.172900	3.690800	-1.350500
H	4.508600	3.140300	-1.611700
H	4.964100	3.891000	-0.061600
H	7.657300	2.456500	0.028500
H	6.574600	2.566900	1.438100
H	7.133700	0.983800	0.848200
H	4.330300	1.545900	1.220700
H	3.648200	1.437700	-0.403200
C	-7.674000	0.553400	1.577300
C	-6.621800	-0.224600	1.089100
C	-6.080900	0.010900	-0.182900
C	-6.623400	1.048600	-0.955400
C	-7.674700	1.829600	-0.471700
C	-8.203900	1.584500	0.798100
H	-8.081600	0.351900	2.564200
H	-6.216800	-1.028000	1.700100
H	-6.220400	1.242200	-1.946900
H	-8.083300	2.626100	-1.087500
H	-9.024000	2.188900	1.174900
C	-4.915300	-0.807700	-0.695100
H	-4.926700	-1.802100	-0.235100
H	-5.012600	-0.957000	-1.777300
C	-3.544400	-0.139900	-0.405000
H	-3.430900	0.006700	0.675200
H	-3.550900	0.860400	-0.861600
C	-2.389800	-0.926400	-0.945600
H	-2.374600	-1.090200	-2.024400
C	-1.387100	-1.444800	-0.197800
H	-1.396300	-1.283800	0.880900
C	-0.294200	-2.222800	-0.737300
H	-0.331900	-2.439400	-1.801000
C	0.730200	-2.779500	0.023800
H	1.372800	-3.536400	-0.416400
H	0.659200	-2.779700	1.112300

==============================

^{2}F’

==============================

Ti	1.486500	-0.835800	-0.101000
Cl	1.187800	0.073100	2.087700
C	0.910800	0.143900	-1.554500
H	0.360000	1.000400	-1.945300
H	1.265600	-0.539400	-2.355400
N	4.692300	2.663800	-0.309900
N	3.739100	-1.045200	-0.425700
C	4.026400	-1.978400	-1.549300
C	4.359100	-1.597800	0.810800
C	4.368300	0.268200	-0.786200
C	4.037800	3.835300	0.270000
C	6.133200	2.716400	-0.068500
C	4.086600	1.422100	0.181500
H	5.109000	-2.047800	-1.722000
H	3.645500	-2.972700	-1.316600
H	3.546400	-1.614500	-2.459600
H	5.447900	-1.680800	0.694200
H	4.135300	-0.958400	1.664500
H	3.951700	-2.592700	1.005600
H	5.450400	0.102800	-0.884200
H	3.978300	0.550700	-1.765800
H	4.472500	4.744600	-0.156800
H	2.970300	3.823500	0.031600
H	4.144800	3.892900	1.369700
H	6.539600	3.640700	-0.490400
H	6.388000	2.691200	1.008400
H	6.641500	1.880300	-0.555700
H	4.426900	1.185100	1.205400
H	3.004300	1.575900	0.231700
C	-6.799400	1.760200	-1.450800
C	-5.537200	1.336400	-1.029800
C	-5.375200	0.641800	0.177900
C	-6.514200	0.383300	0.954900
C	-7.778500	0.805000	0.538300
C	-7.925400	1.495000	-0.667600
H	-6.902600	2.301200	-2.387500
H	-4.664300	1.551000	-1.642100
H	-6.406900	-0.148200	1.897700
H	-8.647500	0.599200	1.157400
H	-8.907700	1.827000	-0.991700
C	-4.011600	0.149500	0.612000
H	-3.233600	0.825400	0.239900
H	-3.943900	0.158500	1.706400
C	-3.706900	-1.285700	0.103700
H	-3.758400	-1.301900	-0.991200
H	-4.503200	-1.950200	0.468900
C	-2.375100	-1.792200	0.567300
H	-2.232200	-1.866500	1.646400
C	-1.351200	-2.137000	-0.248800
H	-1.478400	-2.054300	-1.327400
C	-0.084500	-2.651200	0.223700
H	0.000300	-2.803400	1.300100
C	0.977800	-3.053900	-0.578000
H	0.869400	-3.114800	-1.658100
H	1.771300	-3.652900	-0.138200

==============================

^{2}F-TS

==============================

Ti	2.461600	-1.479400	-0.072400
Cl	0.908200	-1.862500	-1.880300
C	3.936100	-2.677500	0.197100
H	4.023300	-3.707600	-0.176300
H	4.803400	-2.428000	0.827800
N	3.431800	0.487800	-0.704200
N	0.926700	3.314300	-0.067900
C	-0.163900	3.490000	0.889500
C	1.673100	4.561300	-0.223800
C	1.787200	2.186000	0.300800
C	4.079400	0.176100	-2.010300

C	4.497300	0.825300	0.275700
C	2.520600	1.651800	-0.935300
H	0.181700	3.776000	1.901400
H	-0.840600	4.272800	0.533000
H	-0.732000	2.557600	0.973300
H	2.159800	4.888200	0.715000
H	2.447100	4.460500	-0.988700
H	0.992400	5.355100	-0.546000
H	2.495900	2.466000	1.100300
H	1.145400	1.398300	0.701800
H	4.759400	-0.667000	-1.883300
H	3.312800	-0.094600	-2.738900
H	4.636800	1.046900	-2.381800
H	5.221700	0.010100	0.302600
H	5.012300	1.752700	-0.011900
H	4.075700	0.950700	1.274100
H	3.114000	2.455500	-1.393900
H	1.773200	1.336900	-1.668000
C	-6.149300	-1.211300	-1.249800
C	-4.785400	-1.056000	-0.993100
C	-4.317900	-0.007900	-0.186900
C	-5.255800	0.883600	0.354800
C	-6.620700	0.732400	0.101400
C	-7.072600	-0.317400	-0.701900
H	-6.490000	-2.028100	-1.880300
H	-4.072400	-1.753700	-1.426500
H	-4.911700	1.706600	0.977200
H	-7.330300	1.436400	0.527700
H	-8.133700	-0.435500	-0.902400
C	-2.844300	0.129000	0.129500
H	-2.243200	-0.260200	-0.699800
H	-2.585000	1.189300	0.238600
C	-2.445800	-0.631900	1.421800
H	-2.680700	-1.694600	1.290400
H	-3.079000	-0.266800	2.242200
C	-0.996800	-0.471300	1.788000
H	-0.692600	0.511500	2.150400
C	-0.079700	-1.455600	1.709400
H	-0.402300	-2.438600	1.362200
C	1.322500	-1.351000	2.105500
H	1.661300	-0.392000	2.496700
C	2.149600	-2.450200	2.236400
H	1.776600	-3.451100	2.038600
H	3.096900	-2.388300	2.754900

===============================

^{2}G

===============================

Ti	2.739400	-1.068800	-0.118700
Cl	4.528600	-1.141300	-1.720800
C	2.685100	-2.806800	1.045100
H	3.604800	-3.387500	1.160700
H	2.159900	-2.753300	2.006400
C	-6.724100	-0.720800	1.352100
C	-5.557200	-1.239000	0.786100
C	-4.981600	-0.650200	-0.349600
C	-5.610300	0.474800	-0.904100
C	-6.776900	0.997400	-0.342000
C	-7.338200	0.401000	0.789800
H	-7.155700	-1.195700	2.229100
H	-5.088200	-2.115700	1.227100
H	-5.183500	0.941800	-1.788900
H	-7.250100	1.866700	-0.790700
H	-8.247700	0.803500	1.226600
C	-3.693300	-1.186400	-0.934800
H	-3.623500	-2.266300	-0.761100
H	-3.690800	-1.040000	-2.021900
C	-2.431700	-0.507700	-0.338500
H	-2.414300	-0.676300	0.745700
H	-2.534700	0.578200	-0.483400
C	-1.150600	-0.996100	-0.955500
H	-1.024500	-0.803700	-2.023400
C	-0.185000	-1.670600	-0.296100
H	-0.353100	-1.863600	0.768400
C	1.092800	-2.154700	-0.836100
H	1.114600	-2.234900	-1.927700
C	1.753200	-3.356300	-0.083400
H	2.339000	-3.944500	-0.796400
H	0.976300	-4.017400	0.324100
N	2.861300	0.637200	1.393600
N	1.354300	3.374100	-0.762500
C	0.071500	3.458800	-1.458200
C	1.710300	4.670500	-0.186900
C	1.326500	2.308800	0.241200
C	4.237700	0.510600	1.949300
C	1.884000	0.466800	2.502100
C	2.731600	1.997300	0.775700
H	-0.768600	3.723600	-0.788600
H	0.130100	4.220800	-2.241200
H	-0.160300	2.499800	-1.931600
H	1.002600	5.000700	0.597500
H	2.711800	4.639000	0.250000
H	1.717900	5.427900	-0.976200
H	0.646600	2.559600	1.077400
H	0.915700	1.413600	-0.238300
H	4.367900	-0.488700	2.370300
H	4.970700	0.659600	1.153000
H	4.410600	1.257400	2.735000
H	2.080200	-0.479000	3.009000
H	1.975300	1.287900	3.225500
H	0.866500	0.448200	2.109200
H	3.040000	2.741000	1.523100
H	3.440100	2.047900	-0.057200

===============================

^{2}G’

===============================

Ti	-2.709300	-1.220600	-0.032100
Cl	-3.975800	-3.164900	-0.637900
C	-1.086000	-1.750200	1.192700
H	-1.155700	-2.661400	1.795800
H	-0.651000	-0.935900	1.786900

C	7.638700	-0.070500	1.419600
C	6.245800	-0.042600	1.321300
C	5.613500	0.280300	0.111800
C	6.417500	0.576800	-0.999100
C	7.810500	0.550000	-0.906600
C	8.426700	0.225200	0.304600
H	8.108100	-0.319600	2.367400
H	5.639300	-0.270400	2.194900
H	5.945600	0.835200	-1.944500
H	8.414200	0.786500	-1.778600
H	9.510200	0.206500	0.379500
C	4.104700	0.265900	-0.005400
H	3.651200	0.532300	0.956300
H	3.783000	1.025600	-0.728500
C	3.551300	-1.111500	-0.451400
H	3.837300	-1.872700	0.284600
H	4.047700	-1.385400	-1.393700
C	2.059100	-1.110500	-0.646800
H	1.675100	-0.415900	-1.394700
C	1.196500	-1.887500	0.016800
H	1.584300	-2.583600	0.765100
C	-0.305800	-1.925300	-0.152900
H	-0.554600	-2.929300	-0.535400
C	-0.940200	-0.882900	-1.109600
H	-0.895900	-1.143500	-2.172100
H	-0.505700	0.112000	-0.946300
N	-2.121000	3.331400	-0.061100
N	-4.289000	0.275400	0.524500
C	-4.904300	-0.235000	1.780000
C	-5.342000	0.359800	-0.524800
C	-3.696600	1.620800	0.798900
C	-1.345100	3.760000	-1.224100
C	-2.760400	4.481500	0.578100
C	-3.086200	2.297700	-0.438100
H	-5.712900	0.427800	2.113800
H	-5.312900	-1.232700	1.605200
H	-4.145200	-0.290800	2.563700
H	-6.105500	1.097400	-0.245600
H	-4.900100	0.645800	-1.480400
H	-5.811600	-0.618700	-0.637700
H	-4.476500	2.251400	1.246300
H	-2.907400	1.487600	1.546100
H	-0.595600	4.493500	-0.912100
H	-0.823000	2.903200	-1.660500
H	-1.966400	4.223500	-2.013400
H	-1.997400	5.220500	0.839000
H	-3.505900	4.974400	-0.074400
H	-3.261100	4.183300	1.502900
H	-3.884400	2.706200	-1.085800
H	-2.549900	1.544900	-1.027800

================================

^{2}H

================================

Ti	1.885200	-0.848500	0.078400
Cl	0.863200	-0.865200	-2.130400
C	3.859600	-1.815800	-0.147900
H	4.142600	-1.845000	-1.207400
H	4.617400	-1.245400	0.407300
C	-5.076300	-0.668800	-1.614500
C	-3.752600	-0.579400	-1.180200
C	-3.439000	-0.598500	0.187400
C	-4.490200	-0.710200	1.108700
C	-5.816500	-0.799700	0.679400
C	-6.114200	-0.779000	-0.685100
H	-5.298000	-0.647300	-2.678100
H	-2.948600	-0.489200	-1.907100
H	-4.266700	-0.720900	2.173200
H	-6.616600	-0.880500	1.410300
H	-7.145100	-0.843900	-1.021400
C	-1.998700	-0.526900	0.646800
H	-1.433200	0.131700	-0.022000
H	-1.956900	-0.080100	1.648800
C	-1.303500	-1.903600	0.689400
H	-1.355400	-2.363300	-0.304900
H	-1.877100	-2.558200	1.364200
C	0.144000	-1.825000	1.123900
H	0.314200	-1.264000	2.059100
C	1.077100	-2.866700	0.867300
H	0.855800	-3.549300	0.044000
C	2.388600	-2.773200	1.310200
H	2.595500	-2.221500	2.232600
C	3.596900	-3.184000	0.493500
H	3.311300	-3.931400	-0.255800
H	4.416800	-3.606200	1.089000
N	2.684800	1.329000	-0.239400
N	-0.536000	3.311600	0.309800
C	-1.452200	3.419700	1.443200
C	-0.290900	4.631900	-0.270400
C	0.698600	2.634400	0.714400
C	3.555100	1.313400	-1.445500
C	3.508800	1.742200	0.924400
C	1.590000	2.318100	-0.494900
H	-1.045800	4.030900	2.271500
H	-2.389200	3.878800	1.114600
H	-1.680700	2.424200	1.835000
H	0.240200	5.313400	0.421600
H	0.295900	4.552400	-1.188600
H	-1.247900	5.093500	-0.530000
H	1.258800	3.236800	1.454800
H	0.405100	1.707500	1.220900
H	4.359900	0.591000	-1.305700
H	2.961800	1.020300	-2.312200
H	3.991900	2.306800	-1.619400
H	4.359700	1.064600	1.018100
H	3.887300	2.766100	0.798300
H	2.924700	1.694200	1.845400
H	2.055600	3.243500	-0.861900
H	0.969200	1.910200	-1.296800

================================

^{2}H’

==============================

Ti	1.493200	-1.114800	-0.114800
Cl	3.268500	-0.732600	-1.726900
C	2.242600	-2.759700	1.153700
H	3.330500	-2.841400	1.040100
H	2.006200	-2.546100	2.205500
C	-5.153600	-0.282400	1.666700
C	-4.137800	-0.971800	1.000900
C	-3.816500	-0.674400	-0.332000
C	-4.544300	0.333700	-0.982100
C	-5.561200	1.025500	-0.321200
C	-5.868900	0.720100	1.007100
H	-5.390600	-0.532500	2.697200
H	-3.592200	-1.758100	1.518200
H	-4.315100	0.572700	-2.018100
H	-6.116800	1.798700	-0.844800
H	-6.662500	1.254600	1.521300
C	-2.688600	-1.392400	-1.037700
H	-2.598000	-2.416600	-0.660800
H	-2.901900	-1.465900	-2.110100
C	-1.317100	-0.677800	-0.872500
H	-1.138800	-0.578800	0.216500
H	-1.408000	0.349600	-1.241500
C	-0.177000	-1.366200	-1.609400
H	-0.067400	-1.072300	-2.650800
C	0.275800	-2.677200	-1.273000
H	0.940300	-3.161600	-1.990200
C	0.293200	-3.144100	0.033300
H	-0.458500	-2.795100	0.744600
C	1.435100	-3.949500	0.621100
H	1.976600	-4.477900	-0.172100
H	1.121800	-4.693600	1.364700
N	2.291200	0.607000	1.267900
N	1.195800	3.757300	-0.624800
C	0.031400	4.053000	-1.457400
C	1.509000	4.898500	0.233900
C	1.000700	2.519600	0.137600
C	3.679700	0.262300	1.673900
C	1.451100	0.681600	2.492000
C	2.350500	1.935600	0.580100
H	-0.890500	4.227600	-0.870700
H	0.225800	4.951900	-2.050300
H	-0.154700	3.225000	-2.148100
H	0.696100	5.132500	0.947800
H	2.421200	4.716000	0.807300
H	1.680200	5.783500	-0.386100
H	0.335100	2.684800	1.004100
H	0.487800	1.813000	-0.522200
H	3.683800	-0.701900	2.183300
H	4.307100	0.195900	0.784500
H	4.085400	1.026600	2.351600
H	1.535700	-0.258100	3.041900
H	1.777500	1.502300	3.145900
H	0.402800	0.835400	2.230200
H	2.854100	2.641400	1.256500
H	2.979300	1.810900	-0.304100

==============================

^{2}H’’

==============================

Ti	-2.098200	-0.553700	0.171000
Cl	-2.194600	-0.773000	2.575500
C	-4.013200	-1.096500	-0.611800
H	-4.670400	-1.331600	0.238600
H	-4.545900	-0.381500	-1.253400
C	5.116700	-2.405700	-0.089800
C	3.721300	-2.394100	-0.087100
C	3.008900	-1.228800	0.238900
C	3.735900	-0.076900	0.570300
C	5.133800	-0.085000	0.570900
C	5.829500	-1.248800	0.239000
H	5.648500	-3.319200	-0.342300
H	3.177500	-3.303100	-0.333900
H	3.197400	0.831500	0.829400
H	5.677800	0.818500	0.833300
H	6.915800	-1.257600	0.240400
C	1.493900	-1.221000	0.240200
H	1.135900	-2.038700	0.877900
H	1.137900	-0.291900	0.700900
C	0.859700	-1.360800	-1.163500
H	1.314700	-2.227100	-1.667400
H	1.144700	-0.490400	-1.766300
C	-0.664900	-1.498100	-1.153500
H	-1.114300	-1.278200	-2.133300
C	-1.217600	-2.690800	-0.513800
H	-0.562900	-3.237900	0.164500
C	-2.531900	-3.063200	-0.528100
H	-2.833700	-3.878100	0.127000
C	-3.619600	-2.393800	-1.350900
H	-4.458200	-3.088400	-1.497500
H	-3.232800	-2.155700	-2.351600
N	-2.113800	1.726900	-0.121400
N	1.515200	2.938500	-0.128100
C	2.562500	2.878900	-1.148100
C	1.439300	4.289200	0.431300
C	0.244200	2.489000	-0.705000
C	-3.227500	2.094800	0.800400
C	-2.522300	2.105800	-1.500700
C	-0.897500	2.485500	0.317000
H	2.364300	3.551600	-2.003700
H	3.521900	3.164700	-0.707200
H	2.657000	1.858500	-1.529300
H	1.117600	5.044600	-0.310500
H	0.749400	4.324500	1.277800
H	2.427400	4.578800	0.799800
H	-0.035900	3.119300	-1.569900
H	0.396700	1.473100	-1.084500
H	-4.113100	1.500900	0.561100
H	-2.933400	1.895400	1.831300
H	-3.476900	3.158400	0.691400
H	-3.480000	1.634300	-1.725100
H	-2.633900	3.194700	-1.590600

H	-1.787200	1.759300	-2.227500
H	-1.204100	3.518100	0.532500
H	-0.551100	2.038900	1.254300

==============================

[2]H-TS

==============================

Ti	-0.638200	0.361200	-0.393100
C	-2.423800	1.941600	-0.777400
H	-2.130300	1.598600	-1.778700
H	-3.382700	1.567300	-0.428200
C	6.140400	-1.055400	-1.253000
C	4.821700	-0.907200	-0.819100
C	4.526400	-0.256400	0.389000
C	5.593500	0.241900	1.150700
C	6.914900	0.097000	0.721100
C	7.193300	-0.552800	-0.483600
H	6.346400	-1.567100	-2.189400
H	4.005800	-1.302900	-1.420100
H	5.385800	0.744600	2.092700
H	7.726400	0.488100	1.329100
H	8.220300	-0.670100	-0.817900
C	3.091000	-0.071900	0.830500
H	2.497700	-0.942000	0.526900
H	3.046200	-0.018300	1.926200
C	2.430100	1.192500	0.241700
H	2.515200	1.151300	-0.853000
H	3.024200	2.067900	0.556000
C	0.970100	1.343200	0.643700
H	0.839200	1.362000	1.733800
C	0.163700	2.301200	-0.107200
H	0.563300	2.779800	-1.003400
C	-1.199400	2.619500	0.258900
H	-1.508700	2.510200	1.297300
C	-2.129300	3.411300	-0.586200
H	-1.687600	3.936100	-1.431000
H	-2.883100	3.997200	-0.067600
Cl	0.328300	-1.367700	-1.845800
N	-5.223200	-0.371600	-0.280000
N	-1.803500	-0.952800	1.196200
C	-1.275700	-0.587300	2.535500
C	-1.483200	-2.385600	0.954500
C	-3.282300	-0.762900	1.220800
C	-5.749600	-0.563400	-1.630500
C	-6.234100	-0.719500	0.719100
C	-3.980300	-1.125300	-0.099100
H	-1.737500	-1.205800	3.318200
H	-0.195400	-0.735800	2.556200
H	-1.488400	0.462600	2.746800
H	-1.955500	-3.019300	1.718800
H	-1.826000	-2.691100	-0.033200
H	-0.401800	-2.525800	0.992400
H	-3.694900	-1.357800	2.049300
H	-3.475700	0.289000	1.445600
H	-6.647800	0.047200	-1.762800
H	-5.007000	-0.243400	-2.367800
H	-6.017200	-1.614700	-1.845100
H	-7.134700	-0.123400	0.546000
H	-6.518600	-1.788000	0.686200
H	-5.875000	-0.493800	1.726500
H	-4.157300	-2.215800	-0.151700
H	-3.323400	-0.874600	-0.939900

==============================

[4]H-TS

==============================

Ti	0.682700	0.380800	0.498800
Cl	-0.061300	-1.385600	2.128300
N	5.305300	-0.287300	0.225800
N	1.845400	-1.052100	-1.087000
C	1.276400	-0.813200	-2.432500
C	1.588700	-2.470400	-0.737500
C	3.310700	-0.800100	-1.166400
C	5.888900	-0.394700	1.561400
C	6.286600	-0.656700	-0.793700
C	4.080800	-1.087600	0.132100
H	1.729600	-1.481400	-3.181100
H	0.200100	-0.994400	-2.409400
H	1.456100	0.219700	-2.739500
H	2.072700	-3.145600	-1.460300
H	1.956300	-2.690400	0.263900
H	0.512500	-2.650600	-0.744000
H	3.725800	-1.403400	-1.989500
H	3.448300	0.252700	-1.429000
H	6.779700	0.237700	1.623300
H	5.170500	-0.048100	2.310600
H	6.186700	-1.427800	1.821800
H	7.170600	-0.019500	-0.696000
H	6.613100	-1.710800	-0.711900
H	5.877300	-0.505400	-1.795900
H	4.303100	-2.168900	0.209700
H	3.461300	-0.838700	1.001100
C	2.361000	2.117400	0.754200
H	2.173800	1.989700	1.826100
H	3.316700	1.729700	0.408600
C	-6.339300	-0.270100	1.409600
C	-4.989300	-0.214900	1.061300
C	-4.581600	-0.344800	-0.276000
C	-5.566900	-0.534200	-1.254200
C	-6.920700	-0.590400	-0.910900
C	-7.311700	-0.457100	0.422700
H	-6.632600	-0.172700	2.451400
H	-4.238200	-0.077000	1.835900
H	-5.270100	-0.639800	-2.295100
H	-7.668200	-0.740500	-1.685300
H	-8.363000	-0.503400	0.692400
C	-3.116100	-0.237400	-0.642200
H	-2.516200	-0.868100	0.023900
H	-2.962200	-0.615000	-1.660500
C	-2.594700	1.216200	-0.563700
H	-2.790100	1.619200	0.438200
H	-3.199900	1.821800	-1.257500

C	-1.131900	1.378200	-0.896800
H	-0.822700	1.022100	-1.880100
C	-0.325700	2.322800	-0.251300
H	-0.730400	2.846400	0.618000
C	1.049000	2.566000	-0.576000
H	1.422500	2.246200	-1.545400
C	1.946200	3.461000	0.207000
H	1.435500	4.114700	0.915600
H	2.688300	4.014300	-0.367800

================================

^{2}**I**

================================

Ti	1.176500	-0.176300	0.368700
C	2.312100	3.951100	0.381400
H	2.294300	3.844700	1.463900
H	3.272400	4.273400	-0.014200
C	-5.762700	-0.885000	1.193400
C	-4.428800	-0.893400	0.779800
C	-4.011000	-0.147300	-0.332600
C	-4.972500	0.608100	-1.021000
C	-6.307500	0.621100	-0.611700
C	-6.708100	-0.126400	0.498700
H	-6.064100	-1.474000	2.055500
H	-3.700100	-1.489900	1.324300
H	-4.670000	1.189100	-1.889500
H	-7.035500	1.211200	-1.162100
H	-7.746800	-0.120900	0.816800
C	-2.557700	-0.125200	-0.753000
H	-2.092800	-1.091100	-0.519200
H	-2.493700	0.000000	-1.841900
C	-1.742400	1.000500	-0.077900
H	-1.790000	0.859100	1.012100
H	-2.265500	1.953700	-0.270100
C	-0.286900	1.080900	-0.528600
H	-0.191100	1.186900	-1.618600
C	0.669500	1.900900	0.237300
H	0.295400	2.208800	1.225000
C	1.458800	2.997100	-0.423900
H	1.865100	2.736900	-1.401900
C	1.055800	4.458300	-0.294200
H	0.200500	4.686500	0.337000
H	1.167200	5.118100	-1.151800
Cl	0.888000	-1.427300	2.466200
N	3.481800	-0.267900	0.217200
N	1.389200	-1.909100	-1.225500
C	0.675000	-1.673500	-2.501600
C	0.892100	-3.174300	-0.630400
C	2.855100	-2.001500	-1.489400
C	4.129400	-0.095500	1.540600
C	4.066300	0.716400	-0.722600
C	3.699600	-1.663400	-0.262000
H	0.894600	-2.468400	-3.229700
H	-0.399700	-1.648500	-2.318000
H	0.975700	-0.711900	-2.922600
H	1.031100	-4.014600	-1.326200
H	1.415300	-3.387900	0.302000
H	-0.171200	-3.071100	-0.404000
H	3.118800	-3.009100	-1.841100
H	3.092300	-1.313400	-2.304200
H	3.950400	0.921700	1.895000
H	3.694100	-0.796600	2.253800
H	5.213900	-0.264300	1.473800
H	3.912500	1.719400	-0.324700
H	5.142900	0.536500	-0.857500
H	3.578800	0.663700	-1.697400
H	4.761900	-1.823400	-0.500200
H	3.450200	-2.335400	0.564700

================================

^{3}**J**

================================

Ti	0.411400	1.779400	0.115300
Cl	0.913800	3.788800	1.282100
C	1.677400	1.753900	3.985000
H	2.199700	1.469100	4.895000
H	2.136800	2.569200	3.436000
C	0.993200	0.665600	3.192200
H	1.014900	-0.305300	3.688500
C	0.169200	1.681700	3.951300
H	-0.348900	2.439700	3.377000
H	-0.358900	1.340700	4.838300
C	1.107100	0.471700	1.693600
H	0.663300	-0.485900	1.408400
C	2.238700	0.824300	0.896900
H	2.865100	1.616300	1.314700
C	3.049400	-0.234400	0.163600
H	3.779300	0.232300	-0.507800
H	2.407400	-0.872600	-0.453500
C	3.842000	-1.138800	1.146700
H	3.128900	-1.649900	1.803500
H	4.466200	-0.503900	1.787400
C	4.707300	-2.153100	0.431900
C	6.040000	-1.861800	0.104000
C	4.185300	-3.395400	0.039800
C	6.827500	-2.779600	-0.594600
H	6.465600	-0.907200	0.405700
C	4.968000	-4.316900	-0.658900
H	3.156600	-3.643200	0.291800
C	6.293400	-4.011600	-0.979900
H	7.859000	-2.534600	-0.833500
H	4.544900	-5.275100	-0.948200
H	6.904900	-4.728600	-1.520400
Cl	-0.296300	-0.246700	-1.309700
N	-1.099100	3.316200	-1.190400
N	1.731900	2.453200	-1.888100
C	0.827600	3.254400	-2.764900
H	1.428300	3.888500	-3.433200
H	0.285300	2.554400	-3.403900
C	2.867900	3.302700	-1.444700
H	3.534000	2.713700	-0.814000
H	2.505400	4.146900	-0.861300

H	3.430500	3.672000	-2.314400
C	-2.042000	2.583500	-2.068200
H	-2.758700	2.056400	-1.438500
H	-1.522700	1.847000	-2.678400
H	-2.584600	3.281900	-2.723500
C	2.288700	1.372200	-2.741400
H	2.758200	1.802400	-3.638200
H	1.492200	0.692000	-3.040100
H	3.039800	0.811100	-2.192600
C	-1.911400	4.242500	-0.363900
H	-2.496200	4.916000	-1.008000
H	-1.259100	4.832700	0.277800
H	-2.588800	3.662000	0.261900
C	-0.154200	4.121500	-2.000000
H	-0.705200	4.750800	-2.717400
H	0.376200	4.790800	-1.318900
Ti	-2.296900	-1.179900	0.196600
Cl	-1.713200	-2.733200	2.060600
Cl	-2.033900	1.189600	1.218500
N	-2.602200	-2.947500	-1.294300
N	-4.600700	-1.354400	0.187600
C	-4.983300	-2.391500	-0.813700
H	-5.955700	-2.833000	-0.556000
H	-5.108900	-1.898100	-1.779900
C	-5.047600	-1.768100	1.543800
H	-4.741800	-1.015100	2.272900
H	-4.584100	-2.714800	1.818900
H	-6.141600	-1.868400	1.572400
C	-2.604600	-2.481500	-2.701600
H	-1.604100	-2.137400	-2.964700
H	-3.293600	-1.645100	-2.832600
H	-2.898600	-3.292200	-3.383900
C	-5.251700	-0.063000	-0.147600
H	-6.345900	-0.162900	-0.158100
H	-4.921000	0.274000	-1.133200
H	-4.966900	0.686200	0.592800
C	-1.579400	-4.012900	-1.162300
H	-1.800500	-4.854400	-1.834900
H	-1.550600	-4.360300	-0.129900
H	-0.601300	-3.601400	-1.421100
C	-3.933200	-3.491200	-0.915300
H	-4.266400	-4.246200	-1.644200
H	-3.815400	-3.992200	0.049300

References

(1) N. T. Jones, S. T. Liddle, C. Wilson and P. L. Arnold, *Organometallics*, 2007, **26**, 755.

(2) Y. Nishida, N. Hosokawa, M. Murai and K. Takai, *J. Am. Chem. Soc.*, 2015, **137**, 114.

(3) (a) B. Liu, T.-Y. Liu, S.-W. Luo and L.-Z. Gong, *Org. Lett.*, 2014, **16**, 6164; (b) S. M. Jing, V. Balasanthiran, V. Pagar, J. C. Gallucci and T. V. RajanBabu, *J. Am. Chem. Soc.*, 2017, **139**, 18034.

(4) L. F. Walker, A. Bourghida, S. Connolly and M. Wills, *J. Chem. Soc., Perkin Trans. 1*, 2002, 965.

(5) A. A. A. Aguilar, G. N. Ledesma, B. Tirloni, T. S. Kaufman and E. L. Larghi, *Synthesis*, 2019, **51**, 4253.

(6) E. Vedejs, G. P. Meier and K. A. J. Snoble, *J. Am. Chem. Soc.*, 1981, **103**, 2823.

(7) J. H. Sim and C. E. Song, *Angew. Chem., Int. Ed.*, 2017, **56**, 1835.

(8) T. Oshiki, T. Kiriyama, K. Tsuchida and K. Takai, *Chem. Lett.*, 2000, **29**, 334.

(9) S. Matsubara, K. Ukai, T. Mizuno and K. Utimoto, *Chem. Lett.*, 1999, **28**, 825.

(10) (a) G. Zuo and J. Louie, *Angew. Chem. Int. Ed.*, 2004, **43**, 2277; (b) S. Liu, X. Zheng, G. B. Hammand and B. Xu, *Adv. Synth. Catal.*, 2018, **360**, 3667; (c) J. Werth and C. Uyeda, *Chem. Sci.*, 2018, **9**, 1604.

(11) (a) G. M. Sheldrick, *Acta Crystallogr.*, 2008, **A64**, 112. (b) G. M. Sheldrick, *Acta Crystallogr.*, 2015, **C71**, 3.

(12) $R_1 = \Sigma||F_o| - |F_c|| / \Sigma |F_o|$, $wR_2 = [\Sigma w(F_o^2 - F_c^2)^2/\Sigma w(F_o^2)^2]^{1/2}$, $GOF = [\Sigma w(F_o^2 - F_c^2)^2/(n - p)]^{1/2}$;where n = the number of reflections and p = the number of parameters refined.

(13) Gaussian 09, Revision E01, M. J. Frisch, G. W. Trucks, H. B. Schlegel, G. E. Scuseria, M. A. Robb, J. R. Cheeseman, G. Scalmani, V. Barone, B. Mennucci, G. A. Petersson, H. Nakatsuji, M. Caricato, X. Li, H. P. Hratchian, A. F. Izmayloy, J. Bloino, G. Zheng, J. L. Sonnenberg, M. Hada, M. Ehara, K. Toyota, R. Fukuda, J. Hasegawa, M. Ishida, T. Nakajima, Y. Honda, O. Kitao, H. Nakai, T. Vreven, J. A. Montgomery, Jr., J. E. Peralta, F. Ogliaro, M. Bearpark, J. J. Heyd, E. Brothers, K. N. Kudin, V. N. Stagroverov, T. Keith, R. Kobayashi, J. Normand, K. Raghayachari, A. Rendell, J. C. Burant, S. S. Iyengar, J. Tomasi, M. Cossi, N. Rega, J. M. Millam, M. Klene, J. E. Knox, J. B. Cross, V. Bakken, C. Adamo, J. Jaramillo, R. Gomperts, R. E. Stratmann, O. Yazyey, A. J. Austin, R. Cammi, C. Pomelli, J. W. Ochterski, R. L. Martin, K. Morokuma, V. G. Zakrzewski, G. A. Voth, P.

Salvador, J. J. Dannenberg, S. Dapprich, A. D. Daniels, O. Farkas, J. B. Foresman, J. V. Ortiz, J. Cioslowski and D. J. Fox, Gaussian, Inc, Wallingfor CT, 2013.
